# Effects of body size on estimation of mammalian area requirements

**DOI:** 10.1111/cobi.13495

**Published:** 2020-06-18

**Authors:** Michael J. Noonan, Christen H. Fleming, Marlee A. Tucker, Roland Kays, Autumn‐Lynn Harrison, Margaret C. Crofoot, Briana Abrahms, Susan C. Alberts, Abdullahi H. Ali, Jeanne Altmann, Pamela Castro Antunes, Nina Attias, Jerrold L. Belant, Dean E. Beyer, Laura R. Bidner, Niels Blaum, Randall B. Boone, Damien Caillaud, Rogerio Cunha de Paula, J. Antonio de la Torre, Jasja Dekker, Christopher S. DePerno, Mohammad Farhadinia, Julian Fennessy, Claudia Fichtel, Christina Fischer, Adam Ford, Jacob R. Goheen, Rasmus W. Havmøller, Ben T. Hirsch, Cindy Hurtado, Lynne A. Isbell, René Janssen, Florian Jeltsch, Petra Kaczensky, Yayoi Kaneko, Peter Kappeler, Anjan Katna, Matthew Kauffman, Flavia Koch, Abhijeet Kulkarni, Scott LaPoint, Peter Leimgruber, David W. Macdonald, A. Catherine Markham, Laura McMahon, Katherine Mertes, Christopher E. Moorman, Ronaldo G. Morato, Alexander M. Moßbrucker, Guilherme Mourão, David O'Connor, Luiz Gustavo R. Oliveira‐Santos, Jennifer Pastorini, Bruce D. Patterson, Janet Rachlow, Dustin H. Ranglack, Neil Reid, David M. Scantlebury, Dawn M. Scott, Nuria Selva, Agnieszka Sergiel, Melissa Songer, Nucharin Songsasen, Jared A. Stabach, Jenna Stacy‐Dawes, Morgan B. Swingen, Jeffrey J. Thompson, Wiebke Ullmann, Abi Tamim Vanak, Maria Thaker, John W. Wilson, Koji Yamazaki, Richard W. Yarnell, Filip Zieba, Tomasz Zwijacz‐Kozica, William F. Fagan, Thomas Mueller, Justin M. Calabrese

**Affiliations:** ^1^ Smithsonian Conservation Biology Institute National Zoological Park 1500 Remount Road Front Royal VA 22630 U.S.A.; ^2^ Department of Biology University of Maryland College Park MD 20742 U.S.A.; ^3^ Senckenberg Biodiversity and Climate Research Centre Senckenberg Gesellschaft für Naturforschung Senckenberganlage 25 Frankfurt (Main) 60325 Germany; ^4^ Department of Biological Sciences Goethe University Max‐von‐Laue‐Straße 9 Frankfurt (Main) 60438 Germany; ^5^ Department of Environmental Science Institute for Wetland and Water Research Radboud University P.O. Box 9010 Nijmegen GL NL‐6500 The Netherlands; ^6^ North Carolina Museum of Natural Sciences Biodiversity Lab Raleigh NC 27601 U.S.A.; ^7^ Fisheries, Wildlife, and Conservation Biology Program, College of Natural Resources Campus Box 8001 North Carolina State University Raleigh NC 27695 U.S.A.; ^8^ Migratory Bird Center Smithsonian Conservation Biology Institute Washington D.C. 20013 U.S.A.; ^9^ Department of Anthropology University of California, Davis Davis CA 95616 U.S.A.; ^10^ Smithsonian Tropical Research Institute Balboa Ancon 0843‐03092 Republic of Panama; ^11^ Environmental Research Division NOAA Southwest Fisheries Science Center Monterey CA 93940 U.S.A.; ^12^ Departments of Biology and Evolutionary Anthropology Duke University Durham NC 27708 U.S.A.; ^13^ Hirola Conservation Programme Garissa 1774–70100 Kenya; ^14^ Department of Ecology and Evolution Princeton University 106A Guyot Hall Princeton NJ 08544 U.S.A.; ^15^ Department of Ecology Federal University of Mato Grosso do Sul Campo Grande MS 79070–900 Brazil; ^16^ Programa de Pós‐Graduaçao em Biologia Animal, Universidade Federal do Mato Grosso do Sul Cidade Universitária Av. Costa e Silva Campo Grande Mato Grosso do Sul 79070‐900 Brazil; ^17^ Camp Fire Program in Wildlife Conservation, State University of New York College of Environmental Science and Forestry Syracuse NY 13210 U.S.A.; ^18^ Michigan Department of Natural Resources 1990 U.S. 41 South Marquette MI 49855 U.S.A.; ^19^ Mpala Research Centre Nanyuki 555–104000 Kenya; ^20^ University of Potsdam, Plant Ecology and Nature Conservation Am Mühlenberg 3 Potsdam 14476 Germany; ^21^ Natural Resource Ecology Laboratory Colorado State University Fort Collins CO 80523 U.S.A.; ^22^ Department of Ecosystem Science and Sustainability Colorado State University Fort Collins CO 80523 U.S.A.; ^23^ National Research Center for Carnivores Conservation Chico Mendes Institute for the Conservation of Biodiversity Estrada Municipal Hisaichi Takebayashi 8600 Atibaia SP 12952‐011 Brazil; ^24^ Instituto de Ecología, Universidad Nacional Autónoma de Mexico and CONACyT Ciudad Universitaria Mexico D.F. 04318 Mexico; ^25^ Jasja Dekker Dierecologie Enkhuizenstraat 26 Arnhem WZ 6843 The Netherlands; ^26^ Wildlife Conservation Research Unit, Department of Zoology University of Oxford Tubney House, Oxfordshire Oxford OX13 5QL U.K.; ^27^ Future4Leopards Foundation Tehran Iran; ^28^ Giraffe Conservation Foundation PO Box 86099 Windhoek Namibia; ^29^ German Primate Center Behavioral Ecology & Sociobiology Unit Kellnerweg 4 Göttingen 37077 Germany; ^30^ Restoration Ecology, Department of Ecology and Ecosystem Management Technische Universität München Emil‐Ramann‐Straße 6 Freising 85354 Germany; ^31^ The Irving K. Barber School of Arts and Sciences, Unit 2: Biology The University of British Columbia Okanagan Campus, SCI 109, 1177 Research Road Kelowna BC V1V 1V7 Canada; ^32^ Department of Zoology and Physiology University of Wyoming Laramie WY 82071 U.S.A.; ^33^ Zoology and Ecology, College of Science and Engineering James Cook University Townsville QLD 4811 Australia; ^34^ Museo de Historia Natural Universidad Nacional Mayor de San Marcos Lima 15072 Peru; ^35^ Department of Forest Resources Management The University of British Columbia Vancouver BC V6T 1Z4 Canada; ^36^ Bionet Natuuronderzoek Valderstraat 39 Stein 6171EL The Netherlands; ^37^ Norwegian Institute for Nature Research — NINA Sluppen Trondheim NO‐7485 Norway; ^38^ Research Institute of Wildlife Ecology, University of Veterinary Medicine Savoyenstraße 1 Vienna A‐1160 Austria; ^39^ Tokyo University of Agriculture and Technology Tokyo 183–8509 Japan; ^40^ Ashoka Trust for Research in Ecology and the Environment (ATREE) Bangalore Karnataka 560064 India; ^41^ Manipal Academy of Higher Education Manipal Karnataka 576104 India; ^42^ U.S. Geological Survey, Wyoming Cooperative Fish and Wildlife Research Unit, Department of Zoology and Physiology University of Wyoming Laramie WY 82071 U.S.A.; ^43^ Max Planck Institute for Ornithology Vogelwarte Radolfzell Am Obstberg 1 Radolfzell D‐78315 Germany; ^44^ Black Rock Forest 65 Reservoir Road Cornwall NY 12518 U.S.A.; ^45^ Department of Anthropology Stony Brook University Stony Brook NY 11794 U.S.A.; ^46^ Office of Applied Science Department of Natural Resources Rhinelander WI 54501 U.S.A.; ^47^ Institute for the Conservation of Neotropical Carnivores – Pró‐Carnívoros Atibaia Sao Paulo 12945‐010 Brazil; ^48^ Frankfurt Zoological Society Bernhard‐Grzimek‐Allee 1 Frankfurt 60316 Germany; ^49^ Embrapa Pantanal Rua 21 de setembro 1880 Corumb´a MS 79320–900 Brazil; ^50^ San Diego Zoo Institute of Conservation Research 15600 San Pasqual Valley Road Escondido CA 92027 U.S.A.; ^51^ National Geographic Partners 1145 17th Street NW Washington D.C. 20036 U.S.A.; ^52^ Department of Ecology Federal University of Mato Grosso do Sul Campo Grande MS 79070–900 Brazil; ^53^ Centre for Conservation and Research 26/7 C2 Road, Kodigahawewa Julpallama Tissamaharama 82600 Sri Lanka; ^54^ Anthropologisches Institut Universität Zürich Winterthurerstrasse 190 Zurich 8057 Switzerland; ^55^ Integrative Research Center Field Museum of Natural History Chicago IL 60605 U.S.A.; ^56^ Department of Fish and Wildlife Sciences University of Idaho 875 Perimeter Drive MS 1136 Moscow ID 83844‐1136 U.S.A.; ^57^ Department of Biology University of Nebraska at Kearney Kearney NE 68849 U.S.A.; ^58^ Institute for Global Food Security (IGFS), School of Biological Sciences Queen's University Belfast Belfast BT9 5DL U.K.; ^59^ School of Biological Sciences Queen's University Belfast 19 Chlorine Gardens Belfast Northern Ireland BT9 5DL U.K.; ^60^ School of Life Sciences Keele University Keele Staffordshire ST5 5BG U.K.; ^61^ Institute of Nature Conservation Polish Academy of Sciences Mickiewicza 33 Krakow 31–120 Poland; ^62^ 1854 Treaty Authority 4428 Haines Road Duluth MN 55811 U.S.A.; ^63^ Asociación Guyra Paraguay – CONACYT Parque Ecológico Asunción Verde Asuncion 1101 Paraguay; ^64^ Instituto Saite Coronel Felix Cabrera 166 Asuncion 1101 Paraguay; ^65^ Wellcome Trust/DBT India Alliance Hyderabad 500034 India; ^66^ School of Life Sciences University of KwaZulu‐Natal Westville Durban 4041 South Africa; ^67^ Centre for Ecological Sciences Indian Institute of Science Bangalore 560012 India; ^68^ Department of Zoology & Entomology University of Pretoria Pretoria 0002 South Africa; ^69^ Ibaraki Nature Museum Zoological Laboratory 700 Osaki Bando‐city Ibaraki 306–0622 Japan; ^70^ Forest Ecology Laboratory Department of Forest Science Tokyo University of Agriculture 1‐1‐1 Sakuragaoka Setagaya‐Ku Tokyo 156–8502 Japan; ^71^ School of Animal, Rural and Environmental Sciences Nottingham Trent University Brackenhurst Campus Southwell NG25 0QF U.K.; ^72^ Tatra National Park Kúznice 1 Zakopane 34–500 Poland

**Keywords:** allometry, animal movement, area‐based conservation, autocorrelation, home range, kernel density estimation, reserve design, scaling, alometría, autocorrelación, conservación basada en áreas, diseño de reserva, distribución local, escalamiento, estimación de densidad del núcleo, movimiento de mamíferos, 异速增长, 动物移动, 区域保护, 自相关, 家域, 核密度估计, 保护区设计, 标度

## Abstract

Accurately quantifying species’ area requirements is a prerequisite for effective area‐based conservation. This typically involves collecting tracking data on species of interest and then conducting home‐range analyses. Problematically, autocorrelation in tracking data can result in space needs being severely underestimated. Based on the previous work, we hypothesized the magnitude of underestimation varies with body mass, a relationship that could have serious conservation implications. To evaluate this hypothesis for terrestrial mammals, we estimated home‐range areas with global positioning system (GPS) locations from 757 individuals across 61 globally distributed mammalian species with body masses ranging from 0.4 to 4000 kg. We then applied block cross‐validation to quantify bias in empirical home‐range estimates. Area requirements of mammals <10 kg were underestimated by a mean approximately15%, and species weighing approximately100 kg were underestimated by approximately50% on average. Thus, we found area estimation was subject to autocorrelation‐induced bias that was worse for large species. Combined with the fact that extinction risk increases as body mass increases, the allometric scaling of bias we observed suggests the most threatened species are also likely to be those with the least accurate home‐range estimates. As a correction, we tested whether data thinning or autocorrelation‐informed home‐range estimation minimized the scaling effect of autocorrelation on area estimates. Data thinning required an approximately93% data loss to achieve statistical independence with 95% confidence and was, therefore, not a viable solution. In contrast, autocorrelation‐informed home‐range estimation resulted in consistently accurate estimates irrespective of mass. When relating body mass to home range size, we detected that correcting for autocorrelation resulted in a scaling exponent significantly >1, meaning the scaling of the relationship changed substantially at the upper end of the mass spectrum.

## Abstract 2

*Article impact statement*: Due to autocorrelation‐induced bias, conventional methods severely underestimate the area requirements of GPS‐tracked large mammals.

## Abstract (es)

Efectos del Tamaño Corporal sobre la Estimación de los Requerimientos de Área de Mamíferos

**Resumen**

La cuantificación precisa de los requerimientos de área de una especie es un prerrequisito para que la conservación basada en áreas sea efectiva. Esto comúnmente implica la recolección de datos de rastreo de la especie de interés para después realizar análisis de la distribución local. De manera problemática, la autocorrelación en los datos de rastreo puede resultar en una subestimación grave de las necesidades de espacio. Con base en trabajos previos, formulamos una hipótesis en la que supusimos que la magnitud de la subestimación varía con la masa corporal, una relación que podría tener implicaciones serias para la conservación. Para probar esta hipótesis en mamíferos terrestres, estimamos las áreas de distribución local con las ubicaciones en GPS de 757 individuos de 61 especies de mamíferos distribuidas mundialmente con una masa corporal entre 0.4 y 4,000 kg. Después aplicamos una validación cruzada en bloque para cuantificar el sesgo en estimaciones empíricas de la distribución local. Los requerimientos de área de los mamíferos <10 kg fueron subestimados por una media ∼15% y las especies con una masa ∼100 kg fueron subestimadas en ∼50% en promedio. Por lo tanto, encontramos que la estimación del área estaba sujeta al sesgo inducido por la autocorrelación, el cual era peor para las especies de talla grande. En combinación con el hecho de que el riesgo de extinción incrementa conforme aumenta la masa corporal, el escalamiento alométrico del sesgo que observamos sugiere que la mayoría de las especies amenazadas también tienen la probabilidad de ser aquellas especies con las estimaciones de distribución local menos acertadas. Como corrección, probamos si la reducción de datos o la estimación de la distribución local informada por la autocorrelación minimizan el efecto de escalamiento que tiene la autocorrelación sobre las estimaciones de área. La reducción de datos requirió una pérdida de datos del ∼93% para lograr la independencia estadística con un 95% de confianza y por lo tanto no fue una solución viable. Al contrario, la estimación de la distribución local informada por la autocorrelación resultó en estimaciones constantemente precisas sin importar la masa corporal. Cuando relacionamos la masa corporal con el tamaño de la distribución local, detectamos que la corrección de la autocorrelación resultó en un exponente de escalamiento significativamente >1, lo que significa que el escalamiento de la relación cambió sustancialmente en el extremo superior del espectro de la masa corporal.

## 摘要

准确量化物种的栖息面积需求是开展有效区域保护的先决条件, 这一般包括收集目标物种的跟踪数据并进行家域分析。然而, 跟踪数据中的自相关性可能导致严重低估动物的空间需求。基于已有研究, 我们假设低估程度会随体重变化而变化, 这种关系可能对保护产生严重影响。为了评估陆生哺乳动物是否符合这一假设, 我们利用体重在 0.4 到 4000 千克之间的61种全球分布哺乳动物的 757 个个体的全球定位系统 (GPS) 定位数据, 估计了它们的家域范围。接下来, 我们应用分块交叉验证定量分析了家域经验估计的偏差。结果显示, 体重小于 10 千克的哺乳动物的面积需求平均被低估了约 15%, 体重在100千克左右的物种平均被低估约 50% 。因此, 我们指出面积估计受到自相关引起偏差的影响, 且这种偏差对大型物种来说更为严重。考虑到物种灭绝风险随体重增加而增加这一事实, 我们观察到的偏差异速增长标度表明, 对最受威胁物种的家域估计可能也最不准确。为了修正这一偏差, 我们测试了数据抽稀和考虑自相关的家域估计是否能最大程度减少自相关对面积估计的尺度效应。数据抽稀需要丢弃约 93% 的数据才能达到 95% 置信的统计独立性, 因此该方法不可行。比较起来, 考虑自相关的家域估计始终可以获得准确估计, 而与动物体型无关。当将体重与家域大小联系起来时, 我们发现校正自相关会导致标度指数显著大于1, 这意味着相关关系的标度在体型最大的物种中发生了显著的变化。 **【翻译: 胡怡思; 审校: 聂永刚】**

## Introduction

Globally, human‐altered landscapes are restricting animal movement (Fahrig 2007; Tucker et al. 2018), and habitat loss and fragmentation are the principal threats to terrestrial biodiversity (Brooks et al. 2002; Wilson et al. 2016). A key component to conserving species in increasingly human‐dominated landscapes is understanding how much space is required to maintain stable, interconnected populations (Brashares et al. 2001; Pe'er et al. 2014). Area requirements are typically quantified via home‐range analysis (Burt 1943). This routinely involves collecting tracking data on species of interest (Kays et al. 2015) and then applying a home‐range estimator to these data (Fleming et al. 2015; Noonan et al. 2019). These range estimates can then be used to inform recommendations on reserve sizes (Linnell et al. 2001), to advocate for specific land‐tenure systems (Johansson et al. 2016; Farhadinia et al. 2018), and to make conservation policy recommendations (Barton´ et al. 2019). However, tracking data are often strongly autocorrelated, whereas conventional home‐range estimators are based on the assumption of independent and identically distributed data (Noonan et al. 2019).

When data are autocorrelated, the total number of data points does not reflect the total amount of information in the data set (i.e., effective sample size) (Fleming & Calabrese 2017). Although the idea that autocorrelation may affect home‐range estimates is not new (e.g., Swihart & Slade 1985; Fieberg 2007; Fleming et al. 2015), only recent analyses have demonstrated the seriousness of the problem. Using the largest empirical tracking data set assembled to date, Noonan et al. (2019) found conventional estimators significantly negatively biased when used on autocorrelated data. Although any form of bias is undesirable, the systematic underestimation of home‐range areas is a worst‐case scenario from a conservation perspective. Any policy or management decisions informed by underestimated home‐range estimates could result in failed conservation initiatives (Brashares et al. 2001; Gaston et al. 2008) or exacerbate negative human–wildlife interactions at reserve boundaries (Van Eeden et al. 2018).

Noonan et al. (2019) noticed that large‐bodied species tended to exhibit more negatively biased conventional home‐range estimates than small‐bodied species. However, the species included in their study were not selected to provide the broad range of body masses required to investigate allometric trends. We compiled an extensive empirical data set of global positioning system (GPS) locations from 757 individuals across 61 terrestrial mammalian species with body masses ranging from 0.4 to 4000 kg. We used these data to investigate whether the underestimation of home‐range size scales with body mass. To see the potential for this, consider that large species have large home ranges (Jetz et al. 2004) that tend to take longer to cross than smaller home ranges (Calder 1983). In addition, range crossing time (*τ_p_*) interacts with the sampling interval (*dt*) in determining the amount of autocorrelation in tracking data (Fleming & Calabrese 2017; Noonan et al. 2019). When *dt* ≲ *τ_p_*, the resulting data are autocorrelated, whereas *dt* ≫ *τ_p_* results in effectively independent data. Finally, the magnitude of the negative biases in conventional home‐range estimates increases in proportion to the strength of autocorrelation in the data (Noonan et al. 2019). Combining these facts, we arrived at the hypothesis that an allometry in *τ_p_* drives autocorrelation and negative estimation bias to scale with body size.

We examined this hypothesis in 2 ways. First, we tested whether the chain of relationships that would drive bias to scale with mass holds for empirical tracking data. Second, we explored how well 2 methods of home‐range estimation for autocorrelated data eliminate the scaling of home‐range estimation bias. These methods were model‐informed data thinning, which removes autocorrelation from the data prior to home‐range estimation, and autocorrelation‐informed home‐range estimation, which statistically accounts for autocorrelation in movement data. We then used model selection to determine whether significant allometry bias remains in the data for each approach and identified whether one of these corrections offers improved performance over the other. Finally, in light of our findings, we revisited the concept of home‐range allometry (e.g., McNab 1963; Jetz et al. 2004; Tucker et al. 2014). Mammalian home‐range area (*H*) scales positively with body mass (*M*) as *H* = *B*_0_ *M^b^*, where *B*_0_ is a normalization constant and *b* is the scaling exponent (McNab 1963). Despite decades of research, however, there has been little consensus on whether the allometry is linear (i.e., *M* ^1^), or superlinear (i.e., *M^>^* ^1^). Historically, this scaling relationship has been calculated by compiling home‐range areas estimated via conventional estimators, which are subject to varying levels of autocorrelation‐induced bias (Noonan et al. 2019), whereas no one has assessed this relationship directly from tracking data. Although consistent bias across the mass spectrum would lead only to a change in the normalization constant, differential bias across the mass spectrum could alter the scaling exponent, fundamentally changing the properties of the relationship. As such, we tested for any significant deviations from linear (*M* ^1^) scaling.

## Methods

All analyses were based on precollected tracking data sets obtained under appropriate permits and that were based on Institutional Animal Care and Use Committee approved protocols.

### Data Compilation

**Figure 1:**
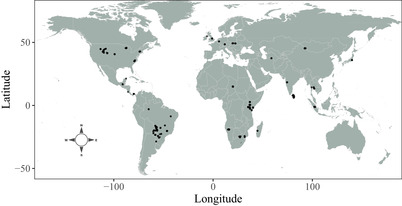
Distribution of study sites for the empirical global positioning system tracking data set spanning 757 individuals across 61 mammalian species.

To investigate whether biases in home‐range estimation scale with body size, we compiled GPS tracking data for 61 globally distributed terrestrial mammalian species, comprising 6.94 × 10^6^ locations for 757 individuals collected from 2000 to 2019 (Fig. 1). Individual data sets were selected based on the criterion of range resident behavior (i.e., area‐restricted space use), as evidenced by plots of the semivariance in positions as a function of the time lag separating observations (i.e., variograms) with a clear asymptote at large lags (Calabrese et al. 2016). When data do not indicate evidence of range residency, home‐range estimation is not appropriate (Calabrese et al. 2016; Fleming & Calabrese 2017), so we excluded data from migratory or nonrange resident individuals. The visual verification of range residency via variogram analysis was conducted using the R package ctmm (version 0.5.3) (Calabrese et al. 2016). Further details on these data are given in Supporting Information.

For each of the species in our data set, we compiled covariate data on that species’ mean adult mass in kilograms. We also identified the main food source for each species and classified them as carnivorous or omnivorous or frugivorous or herbivorous. Data from these 2 dietary classes were analyzed separately. Mass and dietary data were from the EltonTraits database (Wilman et al. 2014).

### Tracking‐Data Analyses

Our conjecture that the underestimation of home‐range areas increases as body size increases was based on 2 well‐established biological and one methodological relationship: the positive correlation between body mass and home‐range area (Jetz et al. 2004); the positive correlation between home‐range area and range crossing time, *τ_p_* (Calder 1983); and the negative correlation between range crossing time and the effective sample size for area estimation, *N*_area_ (i.e., equivalent number of statistically independent locations [Noonan et al. 2019]). We hypothesized that these conspire to drive 2 previously untested relationships: a potential negative correlation between body mass and *N*_area_ and a potential negative correlation between body mass and home‐range estimator accuracy.

Testing for these relationships first required estimating the autocorrelation structure in each of the individual tracking data sets. To accomplish this, we fitted a series of range‐resident, continuous‐time movement models to the data with the estimation methods developed by Fleming et al. (2019). The fitted models included the independent and identically distributed process, which features uncorrelated positions and velocities; the Ornstein–Uhlenbeck (OU) process, which features correlated positions but uncorrelated velocities (Uhlenbeck & Ornstein 1930); and an OU‐foraging (OUF) process, featuring both correlated positions and velocities (Fleming et al. 2014). We used model selection to identify the best fitting model given the data (Fleming et al. 2014) from which *τ_p_* and *N*_area_ were extracted. To fit and select the movement models, we used the R package ctmm and applied the workflow described by Calabrese et al. (2016).

We estimated home‐range areas for each of the 757 individuals in our tracking database via kernel density estimation (KDE) with Gaussian reference function bandwidth optimization because this is one of the most commonly applied home‐range estimators in ecological research (Noonan et al. 2019). The KDE home ranges were estimated via the methods implemented in ctmm, and the further small‐sample‐size bias correction that was introduced in area‐corrected KDE (Fleming & Calabrese 2017).

Our primary aim was to determine the extent to which autocorrelation‐induced bias in conventional home‐range estimation might increase with body size. This required an objective and statistically sound measure of bias. We applied the well‐established technique of block cross‐validation (Noonan et al. 2019) to quantify bias in empirical home‐range estimates.

By determining the extent to which the results of an analysis generalize to a statistically independent data set, cross‐validation is an effective tool for quantifying bias (Pawitan 2001). For this approach, each individual data set was split in half, and a home‐range area was estimated from the first half of the data only (i.e., training set). Next, the percentage of observations in the second half of the data (i.e., held‐out set) that fell within the specified contour (here 50% and 95%) of the estimated home range was calculated. If the percentage of points included came out consistently higher or lower than the specified contour, then it would suggest positive or negative bias, respectively.

As a further measure of bias, we identified the contour of the home range estimated from the training set that contained the desired percentage of locations in the held‐out set (i.e., 50% and 95%) and compared the area within that contour to the estimated area at the specified quantile. For example, consider that the 95% area estimated on the training data contained only 90% of the locations in the held‐out set, whereas the 97% contour contained 95% of the locations. To measure bias, we would take the ratio between the 97% area and the 95% area. Cross‐validating home‐range estimates in this way can also be seen as providing a measure of how well a home‐range estimate can be expected to capture an animal's future space use, assuming no substantial changes in movement behavior.

Block cross‐validation is based on the assumption that data from the training and held‐out sets are generated from the same processes. To confirm this assumption, we used the Battacharryya distance implementation in ctmm (Winner et al. 2018) as a measure of similarity (range 0–∞) between the mean area and covariance parameters of movement models fitted to the training and held‐out data sets and determined whether the confidence intervals on this distance contained 0 (details are given in Appendix S1 in Noonan et al. [2019]). Using this method, we determined that 160 of 757 individuals had movement models with significantly different parameter estimates between the first and second halves of the data, so we excluded these from our cross‐validation analyses. We found no significant relationship between whether or not a data set was excluded from our analyses and which species the data were from (*p* = 0.52) or between exclusion and how long an individual was tracked (*p* = 0.39). This confirmed that the subsampling required to meet the assumptions of half‐sample cross‐validation did not bias our sample.

### Correction Factors

We explored 2 potential solutions to the allometric scaling of autocorrelation and home‐range estimation bias: thinning data to minimize autocorrelation and using autocorrelation‐informed home‐range estimation.

Conventional kernel methods are based on an assumption of independence; however, they can provide accurate estimates for autocorrelated processes when the sampling is coarse enough that the data appear uncorrelated over time (Hall & Hart 1990). Thus, data thinning presents a potentially straightforward solution to autocorrelation‐induced bias, but requires a balance between reducing autocorrelation and retaining sample size. We, therefore, explored model‐informed data thinning as a means of mitigating size‐dependent home‐range bias. As noted above, the parameter *τ_p_* relates to an individual's range‐crossing time and quantifies the time scale over which positional autocorrelation decays to insignificance. More specifically, because positional autocorrelation decays exponentially at rate 1*/τ_p_*, the time required for the percentage of the original velocity autocorrelation to decay to *α* is *τ_α_* = *τ_p_*ln(1*/α*). Conventionally, data are thinned to independence with a 95% level of confidence, and approximately3*τ_p_* is the time it takes for 95% of the positional autocorrelation to decay. Consequently, we thinned each individual's tracking data to a sampling frequency of *dt* = 3*τ_p_*. We then used autocorrelation functions to quantify how much autocorrelation remained in the thinned data and evaluated the performance of KDEs on these thinned data.

As opposed to manipulating the data to meet the assumptions of the estimator, the second potential solution was to use an estimator that explicitly modeled the autocorrelation in the data. Autocorrelated‐KDE (AKDE) is a generalization of Gaussian reference function KDE that conditions upon the autocorrelation structure of the data when optimizing the bandwidth (Fleming et al. 2015). Following the workflow described by Calabrese et al. (2016), AKDE home‐range areas were estimated conditioned on the selected movement model for each data set, via the methods implemented in ctmm, with the same small‐sample‐size bias correction applied to the conventional KDE area estimates (Fleming & Calabrese 2017). The AKDE is available via the web‐based graphical user interface at ctmm.shinyapps.io/ctmmweb/(Dong et al. 2017).

### Correction Factor Performance

To test for body‐size‐dependent biases in cross‐validation success, we fitted 3 regression models to the cross‐validation results as a function of log_10_‐scaled mass. The models included an intercept‐only model (i.e., no change in bias with mass), linear model, and logistic model. We then identified the best model for the data via small‐sample‐size corrected quasi‐Akaike information criterion (Burnham et al. 2011).

Species may exhibit similarities in traits due to phylogenetic inertia and the constraints of common ancestry; thus, controlled comparisons are required (Harvey & Pagel 1991). Accordingly, we did not treat species data records as independent; rather, we used the phylogenetic distances among species to construct a variance–covariance matrix and defined the correlation structure in our allometric regressions with the R package nlme (version 3.1‐137) (Pinheiro et al. 2018). Phylogenetic relationships between eutherian mammalian orders were based on genetic differences and taken from Liu et al. (2001). Intraorder relationships were taken from more targeted studies aimed at resolving species‐level relationships, including Price et al. (2005) for Artiodactyla, Matthee et al. (2004) for Lagomorpha, Steiner and Ryder (2011) for Perissodactyla, Barriel et al. (1999) for Proboscidea, Perelman et al. (2011) for Primates, and Agnarsson et al. (2010) for Carnivora. For Canidae, however, we took relationships from Lindblad‐Toh et al. (2005), due to better coverage of the species in our data set. The phylogenetic tree was built with the R package ape (version 5.2) (Paradis & Schliep 2019), and branch lengths were computed following Grafen (1989). Phylogenies are given in Supporting Information.

## Results

### Allometric Scaling of Bias

**Figure 2:**
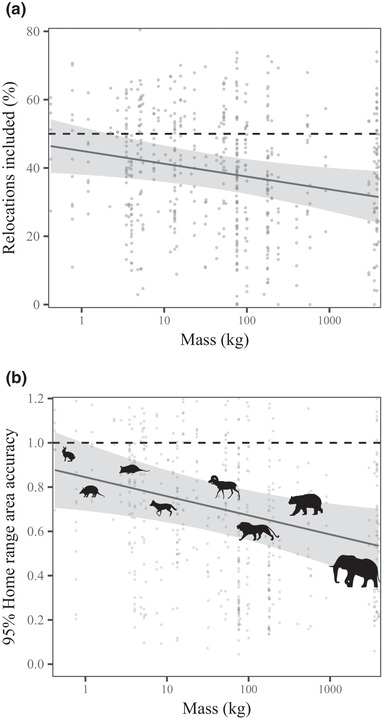
Cross‐validation of conventional kernel density estimation (KDE) across the mammalian body‐mass spectrum: (a) percentage of locations from the second half of the data (held‐out set) included in KDE 50% home ranges estimated from the first half of the data (training set) as a function of body mass (dashed line, target 50% quantile; solid line, phylogenetically controlled regression model fit to cross‐validation results; shading, 95% CI of the fit) and (b) regression model describing the accuracy of 95% KDE area estimates across the mass spectrum. Accuracy was quantified as the ratio between estimated 95% area of the training set and the area contained within the contour that encompassed 95% of locations in the held‐out set. The horizontal dashed line represents an unbiased area estimate. The *x*‐axes in are log scaled.

Out of 757 data sets, only one was independent and identically distributed and free from significant autocorrelation. Conventional KDE 95% home‐range areas cross‐validated at a median rate of 88.3% (95% CI 87.2–90.1), which was below the target 95% quantile and demonstrated a tendency to underestimate home‐range areas on average. Similarly, KDE 50% home‐range areas cross‐validated at a median rate of 41.5% (95% CI 39.4–43.3), which was again below the target 50% quantile. The magnitude of KDE's underestimation worsened as body mass increased (*t* = 2.30, *p* = 0.02) (Fig. 2a), carnivores and herbivores did not differ significantly (*t* = 0.31; *p* = 0.75). Cross‐validation success of 50% home‐range areas across the mass spectrum was best described by a linear decay model with an intercept of 47.2 (95% CI 39.9–54.5) and a slope of –3.9 (95% CI –7.0 to –0.8). In other words, for every order of magnitude increase in body mass, home‐range estimates captured approximately4% less of an individual's future space use.

When comparing the 95% area estimates with the area estimates for the contours that contained 95% of locations, KDE accuracy across the mass spectrum was best described by linear decay (Fig. 2b). Consequently, whereas the home‐range areas of mammals weighing <10 kg were underestimated by 13.6% (95% CI 6.3–18.6), those of species weighing *>*100 kg were underestimated by 46.0% on average (95% CI 36.7–51.4).

### Mechanisms Driving Body Size‐Dependent Estimation Bias

**Figure 3:**
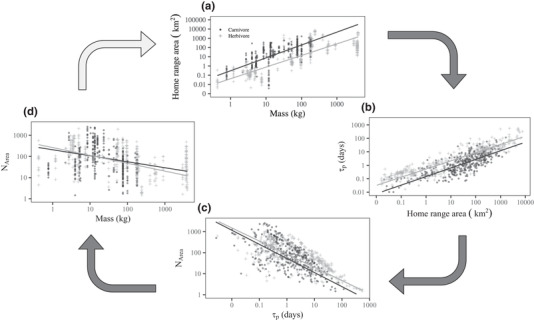
Mechanisms driving body‐size‐dependent estimation bias: (a) positive allometry of home‐range areas, (b) correlation between home‐range area and range‐crossing time (*τ_p_*), (c) negative correlation between *τ_p_* and effective sample size (*N*_area_) governed by duration of observation period (*T*) and *τ_p_* such that *N*_area_ ≈ *T*/*τ_p_*, and (d) resulting negative allometry of *N*_area_ (axes, log scaled; lines, phylogenetically controlled fitted regression models). From (a) to (d), 1 axis is preserved from the previous panel to demonstrate the inherent link between each of these relationships (arrows, visual aid of link; top‐left arrow, end of the chain).

We found significant positive relationships between body mass and home‐range area (regression parameter: *β* = 1.18, 95% CI 0.92–1.43, *t* = 9.09, *p <*0.0001) (Fig. 3a) and between home‐range area and range crossing time, *τ_p_* (*β* = 7.09, 95% CI 4.78–9.41, *t* = 6.00, *p <* 0.0001) (Fig. 3b) and a negative relationship between *τ_p_* and the effective sample size, *N*_area_ (*β* = −0.65, 95% CI –0.70 to –0.60, *t* = 25.46, *p <*0.0001) (Fig. 3c). The former 2 scaling relationships differed significantly between carnivorous and herbivorous mammals (*t* = 3.08, *p <*0.005 and *t* = 2.37, *p* = 0.02, respectively). Carnivores tended to have larger home ranges and shorter range crossing times than comparably sized herbivores, and herbivores tended to have longer range crossing times. The relationship between *N*_area_ and mass did not differ between dietary classes (*t* = 0.82, *p* = 0.06). The *N*_area_ was governed by both *τ_p_* and sampling duration, *T*, such that *N*_area_ ≈ *T*/*τ_p_*. Although we noted a positive correlation between body mass and *T* in the studies we sampled (*β* = 0.24, 95% CI 0.09–0.39, *t* = 3.17, *p* < 0.005), this was not enough to counter the positive correlation between mass and *τ_p_*. Consequently, the net result was a negative relationship between body mass and *N*_area_ (*β* = *–*0.23, 95% CI –0.39 to –0.08, *t* = 2.98, *p <* 0.005) (Fig. 3d).

### Correction Factors

**Figure 4:**
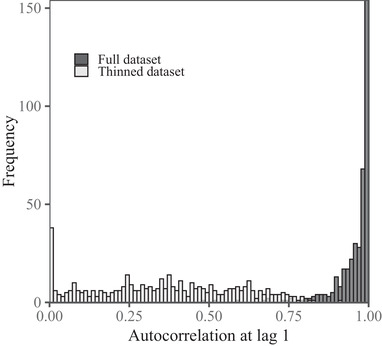
Frequency of amounts of autocorrelation at lag 1 in the full tracking data sets for each of the 757 individuals used to estimate home ranges via conventional kernel density estimation (KDE), compared with the thinned data sets for individuals for which sufficient data remained after thinning to apply KDE.

Model‐informed data thinning served to reduce the mean autocorrelation at lag 1 from 0.96 (95% CI 0.96–0.97) to 0.32 (95% CI 0.30–0.35) (Fig. 4). Hence, an independent and identically distributed model was the best fit for 167 of the 463 individuals for which sufficient data (*>*2 locations) remained after data thinning. The remaining individuals were best described by OU and OUF processes whose autocorrelation parameters were not significant. Although thinning mitigated the correlation between bias and body mass (*β* = –2.41, 95% CI –6.08 to 1.26, *t* = 1.29, *p* = 0.20), the median cross‐validation rate of 95% home ranges estimated using the thinned data was only 85.1% (95% CI 83.6–86.5). This approximately3% decrease in performance, as compared with conventional KDE on the full data, was likely the result of the small sample size. Model‐informed data thinning resulted in a mean data loss of 93.2% (95% CI 92.1–94.3), and the median number of approximately independent locations left in each data set after thinning was only 23 (95% CI 18–26). Furthermore, in approximately20% of the individuals, ≤2 locations remained after thinning, making it impossible to estimate a home‐range area on the thinned data.

### Autocorrelation‐Informed Home‐Range Estimation

Like model‐informed data thinning, autocorrelation‐informed home‐range estimation via AKDE also eliminated the correlation between cross‐validation success and body mass (*β* = –0.51, 95% CI –1.88 to 0.86, *t* = 0.73, *p* = 0.47). However, without the data loss required by the thinning approach, AKDE resulted in a median cross‐validation rate of 95.2% (95% CI 94.2–95.9) for 95% home ranges and 51.3% (95% CI 49.26–54.36) for 50% home ranges. In other words, AKDE exhibited consistent accuracy across species, irrespective of the allometries in autocorrelation time scales and effective sample sizes.

### Scaling of Mammalian Space Use

When regressing home‐range area against mass with conventional KDE estimates, we documented no significant difference from linear scaling for either herbivores or carnivores (Table 1). For AKDE‐derived area estimates, however, we detected that the scaling exponent was significantly >1 for both taxonomic groups, suggesting home‐range area scales with mass according to a power function.

**Table 1:** Estimates of the scaling exponent (*b*) of mass to home‐range area relationship^*^

Category	KDE (95% CI)	AKDE (95% CI)
All mammals	1.20 (0.95–1.45)	1.28 (1.01–1.54)
Herbivores and frugivores	1.26 (0.99–1.52)	1.38 (1.09–1.66)
Carnivores and omnivores	1.23 (0.95–1.50)	1.27 (1.01–1.56)

^*^Abbreviations: KDE, kernel density estimation; AKDE, autocorrelated‐kernel density estimation.

## Discussion

The importance of autocorrelation in animal‐tracking data has been an active area of research for decades (Swihart & Slade 1985; Fieberg 2007; Fleming et al. 2015). We, however, are the first to demonstrate that mass‐specific space requirements driven by autocorrelation‐induced underestimation of home‐range areas are worse for larger species. From a fundamental perspective, the continuous nature of animal movement means quantities, such as positions, velocities, and accelerations, are necessarily autocorrelated (Fleming et al. 2014). Autocorrelation time scales (*τ*) should, therefore, be viewed as explicit attributes of an animal's movement process (Gurarie & Ovaskainen 2015) that are revealed when the temporal resolution of measurement becomes ≲*τ*. As technological advances continue to permit ever‐finer sampling (Kays et al. 2015), persistent autocorrelation is likely to become the norm in animal‐tracking data. Pairing data from inherently autocorrelated processes with statistical approaches that ignore autocorrelation not only risks biasing any derived quantities, but also effectively negates the technological advances that are improving data quality. Unless analyses that are informed by autocorrelation become adopted by movement ecologists and conservationists, the issue of autocorrelation‐induced bias will only worsen. Conversely, properly harnessing the wealth of information provided by autocorrelation can dramatically improve the accuracy of tracking‐data‐derived measures (see also Fleming & Calabrese 2017; Winner et al. 2018; Noonan et al. 2019). Our findings, therefore, highlight the need for more statistical estimators that can handle biologically induced variance without introducing bias.

### Implications of Size‐Dependent Bias

From a conservation perspective, the underestimation of home‐range areas is a worst‐case scenario. When reserves are too small, relative to their target species’ area requirements, the probability of local populations undergoing declines or extirpations increases significantly (Brashares et al. 2001; Gaston et al. 2008). Undersized protected areas resulting from poorly estimated space needs also risk exacerbating the issue of negative human–wildlife interactions at reserve boundaries (Van Eeden et al. 2018) as animals move beyond reserve boundaries to meet their energetic requirements (Farhadinia et al. 2018). It is thus of critical importance that policy actions be well informed about species’ spatial requirements. To this end, we analyzed a broad taxonomic and geographic range of data and identified a strong correlation between home‐range underestimation and body size when autocorrelation was ignored; average bias was approximately 50% at the upper end of the mass spectrum. In this regard, the majority of home ranges are estimated via methods based on the assumption of statistically independent data (Noonan et al. 2019). Combined with the facts that humans are the dominant mortality source for terrestrial vertebrates globally (Hill et al. 2019), that this mortality is higher for large‐bodied species (Hill et al. 2020), and that megafauna are experiencing more severe range contractions (Tucker et al. 2018) and extinction risk (Cardillo et al. 2005), the most threatened species are also likely to be those with the least accurate home‐range estimates, a worrying combination.

Based on these findings, we suggest that any conservation initiatives or policy based on home‐range estimates derived from estimators based on the assumption of statistically independent data be revisited, especially where large‐bodied species are involved. To facilitate this, we developed HRcorrect, an open‐access application that allows users to correct a home‐range area estimate for their focal species’ body‐mass‐specific‐bias with a correction factor calculated from our cross‐validation regression models. The current version of HRcorrect is freely available from https://hrcorrect.shinyapps.io/HRcorrect/. However, there are numerous factors beyond body mass that influence an individual's home‐range size. For instance, mammalian home‐range areas are well known to covary with the spatial distribution of resources (Litvaitis et al. 1986; Boutin 1990), social structure (Lukas & Clutton‐Brock 2013), sex (Cederlund & Sand 1994; Lukas & Clutton‐Brock 2013; Noonan et al. 2018), age (Cederlund & Sand 1994), population density (Adler et al. 1997), and reproductive status (Rootes & Chabreck 1993; Noonan et al. 2018). Furthermore, if an individual's space use changes over time (e.g., interseasonal and ‐annual variation), a home‐range area estimated from a single observation period may not be representative of its long‐term area requirements. As such, the deterministic trend‐based correction provided by HRcorrect is not a substitute for more rigorous data collection and home‐range estimation and should only be used for cases where the underlying tracking data are not accessible.

### Allometries and Conservation Theory

The metabolic theory of ecology (West et al. 1997) suggests that body mass represents a super trait that governs a wide range of ecological processes. Prime among these is the relationship between body mass and home‐range area, an allometry that has guided ecological theory for more than 50 years (McNab 1963; Calder 1983; Jetz et al. 2004). More recently, attempts have been made to integrate this allometry into conservation theory. For instance, Hilbers et al. (2016) incorporated the home‐range allometry into a method for quantifying mass‐specific extinction vulnerability, and Hirt et al. (2018) highlighted how allometries in movement and space use can be used to make testable predictions of movement and biodiversity patterns at the landscape scale. Similarly, Pereira et al. (2004) used allometries of space use and movement rates to predict species‐level vulnerability to land‐use change. If the underlying allometries are biased, however, hypothesis testing and conservation planning in this context can fail even if the logic behind the experimental design is perfectly sound. Although the earliest derivation of the home‐range allometry proposed a metabolically determined *M* ^0^ *^.^* ^75^ allometry (McNab 1963), subsequent revisions showed no support for a purely energetic basis for home‐range scaling (Calder 1983; Kelt & Van Vuren 2001; Jetz et al. 2004; Tucker et al. 2014; Tamburello et al. 2015). Although all these studies concluded that home‐range area should scale with an exponent greater than the 0.75 predicted by metabolic requirements alone, there has been little consensus on whether the allometry is linear (*M* ^1^) or superlinear (*M^>^* ^1^). Our results suggest that at least part of the confusion can be attributed to the increasing bias in underestimating home ranges with increasing body size. Ours is the first study to estimate this relationship directly from tracking data by applying a consistent estimator across all individuals and, crucially, correcting for any potential autocorrelation‐induced bias (Noonan et al. 2019). In doing so, we documented a super‐linear relationship between body mass and home‐range area (exponent of approximately 1.25 for *M*). This shift from linear to power‐law scaling fundamentally changes the behavior of the relationship, particularly at the upper end of the mass spectrum. Although we did not investigate the mechanisms behind the deviation from the metabolically determined *M* ^0^ *^.^* ^75^, we encourage future work on this subject be based on the assumption of a superallometry, as opposed to linear allometry. Accurately quantifying species’ area requirements is a prerequisite for successful, area‐based conservation planning. Our results highlight an important yet hitherto unrecognized aspect of home‐range estimation: autocorrelation‐induced negative bias in home‐range estimation that is systematically worse for large species. Crucially, however, our findings also outline a readily applicable solution to the problem of size‐dependent bias. We demonstrated that home‐range estimation that properly accounts for the autocorrelation structure of the data is currently the only consistently reliable solution for eliminating allometric biases in home‐range estimation (see also Noonan et al. 2019). We emphasize that the differential scaling of autocorrelation across the mass spectrum be a key consideration for movement ecologists and conservation practitioners and suggest avoiding home‐range estimators that assume statistically independent data.

## Supporting information

Data set summary statistics (Appendix S1), individual tracking data set summaries (Appendix S2), and mammalian phylogenetic relationships (Appendix S3) are available online. The authors are solely responsible for the content and functionality of these materials. Queries (other than absence of the material) should be directed to the corresponding author.

Supplementary Material

Supplementary Material
