# Supplementary material for: Effects of body size on estimation of mammalian area requirements
Source: Conserv Biol. 2020 Jun 18;34(4):1017–28. doi: 10.1111/cobi.13495 (PMC7496598; doi:10.1111/cobi.13495)
Supplement: Supplementary file 1 — Data set summary statistics (Appendix S1), individual tracking data set summaries (Appendix S2), and mammalian phylogenetic relationships (Appendix S3) are available online. The authors are solely responsible for the content and functionality of these materials. Queries (other than absence of the material) should be directed to the corresponding author. [file COBI-34-1017-s001.pdf]

Supporting Information S1 — Dataset summary statistics

Table S1.1: Summary statistics of the tracking data used in the analyses in the main text. Values reported are medians. N_{Area} denotes the effective sample size. In the diet column, H/F refers to Herbivorous/Frugivorous species while C/O to Carnivorous/Omnivorous species.

Species	n (individuals)	Frequency (min)	Year	Duration (Days)	n (locations)	n_{Area}	Mass (kg)	Diet	Data Source
Acinonyx jubatus	2	0.02	2012	19.1	104612	39.9	14.2	C/O	MovebankID: 11212867
Aepyceros melampus	21	20	2011	295.5	19623	147.3	52.5	H/F	(Noonan & et al., 2018)
Alces alces	11	60	2009	719	11400	16.6	541.46	H/F	Contributed by co-authors
Antidorcas marsupialis	7	60	2009	227.9	5473	26.7	31.5	H/F	(Abrahms, 2017)
Antilocapra americana	3	480	2002	687.5	1212	35.7	46.08	H/F	Contributed by co-authors
Ateles geoffroyi	3	0.02	2018	169.9	125302	46.5	5.28	H/F	MovebankID: 468460067
Beatragus hunteri	4	60	2014	843.7	20052.5	91	79.13	H/F	(Noonan & et al., 2018)
Brachylagus idahoensis	11	15	2015	13.2	218	76.1	0.42	H/F	(McMahon et al., 2017)
Canis aureus	2	15	2018	244.9	8141.5	1767.4	10.35	C/O	MovebankID: 637111097
Canis latrans	18	15	2010	210.2	10169.5	236.4	13.41	C/O	(Noonan & et al., 2018)
Canis latrans	25	180	2011	400.2	3046	218.1	13.41	C/O	MovebankID: 11521955
Canis lupus	5	15	2010	207	9608	121.6	32.18	C/O	(Noonan & et al., 2018)
Canis lupus familiaris	14	20	2010	15.4	955.5	466.5	12	C/O	Contributed by co-authors
Canis mesomelas	20	60	2009	266.5	6412.5	433.7	8.5	C/O	(Abrahms, 2017)
Cebus capucinus	8	0.02	2018	87.4	92418.5	103.6	2.73	C/O	MovebankID: 468460067
Cerdocyon thous	20	4.93	2012	41	8279.5	116.9	5.24	C/O	(Noonan & et al., 2018)
Cervus canadensis	10	120	2010	734.9	11903	36.9	200	H/F	Contributed by co-authors
Chlorocebus pygerythrus	11	15	2014	329.2	31406	425.6	4.99	H/F	MovebankID: 17629305
Chrysocyon brachyurus	13	120	2010	293.3	2565	410.3	23.25	C/O	MovebankID: 18156143
Connochaetes taurinus	28	60	2011	645.1	8320.5	48.2	180	H/F	MovebankID: 208413731
Cuon alpinus	4	360	2016	163	317	49.3	14.17	C/O	Contributed by co-authors
Dasyprocta punctata	1	15	2009	7.6	214	129.3	2.67	H/F	MovebankID: 8191213
Didelphis virginiana	1	15	2013	48.3	242	51.1	2.2	C/O	MovebankID: 11948467
Elephas maximus maximus	52	240	2011	514.9	2227	52.4	3750	H/F	Contributed by co-authors
Elephas maximus sumatranus	8	120	2014	687.8	6181.5	11.3	3000	H/F	Contributed by co-authors
Equus hemionus hemionus	10	13	2010	362.8	34740	2	240	H/F	Contributed by co-authors
Equus quagga	6	60	2010	328.5	7849	10.4	400	H/F	(Abrahms, 2017)
Erinaceus europaeus	14	20	2013	22.2	137.5	26.5	0.77	C/O	Contributed by co-authors
Eulemur rufifrons	4	30	2009	76.4	4018.5	200.9	2.25	C/O	(Noonan & et al., 2018)
Euphractus sexcinctus	9	5	2014	12.4	340	35.5	4.78	C/O	Contributed by co-authors
Felis silvestris	5	120	2014	82.6	391	93.5	5.1	C/O	MovebankID: 40386102
Giraffa camelopardalis reticulata	7	60	2017	229.1	5476	27.6	899.99	H/F	Contributed by co-authors
Hyaena brunnea	9	156	2009	103.7	244	61	42.98	C/O	Contributed by co-authors
Leopardus pardalis	8	26	2003	346.6	279	93.8	11.9	C/O	MovebankID: 123413
Lepus europaeus	12	60	2014	196.3	2823.5	693.5	3.74	H/F	(Noonan & et al., 2018)
Lepus timidus	1	1	2009	2.6	2594	617.5	3.05	H/F	MovebankID: 2151381
Loxodonta africana	8	60	2009	541.7	12705	5.4	3940.03	H/F	(Abrahms, 2017)
Lynx rufus	1	300	2019	87.6	362	89.2	8.9	C/O	Contributed by co-authors
Madoqua guentheri	15	10	2011	24.9	2194	132.6	7.5	H/F	(Noonan & et al., 2018)
Martes pennanti	14	10	2011	21.6	1425	23.6	4	C/O	MovebankID: 2964494
Nasua narica	2	18	2010	27.4	600.5	34.4	4.03	C/O	(Kays & Hirsch, 2015)
Nasua narica	6	0.02	2017	96.6	100796.5	110.9	4.03	C/O	Movebank ID: 468460067
Odocoileus hemionus	1	180	2012	792.9	5196	655.6	54.21	H/F	Contributed by co-authors
Odocoileus virginianus	5	60	2018	380.6	9117	441.5	55.51	H/F	Contributed by co-authors
Odocoileus virginianus	6	61	2018	170.1	2910	194.8	55.51	H/F	Contributed by co-authors
Oryx dammah	49	60	2017	568.4	11323	39.2	177.5	H/F	Contributed by co-authors
Ovis canadensis	5	420	2006	1030.7	2808	472.6	74.64	H/F	Contributed by co-authors
Panthera leo	3	420	2005	627.8	396	87.7	161.5	C/O	(Noonan & et al., 2018)
Panthera onca	91	240	2012	184.6	615	31.4	75.7	C/O	(Morato et al., 2018)
Panthera pardus pardus	3	15	2014	360	21166	73.1	48.75	C/O	MovebankID: 17629305
Panthera pardus saxicolor	5	60	2015	361.6	3632	68.8	52.04	C/O	Contributed by co-authors
Papio anubis	6	15	2014	306.8	26597.5	137.3	17.73	C/O	MovebankID: 17629305
Papio cynocephalus	21	60	2009	297.5	4098	463.8	15.82	C/O	(Noonan & et al., 2018)
Pecari tajacu	2	103	2016	263.6	223	68.5	21.27	C/O	Contributed by co-authors
Pecari tajacu	1	0.02	2016	28.9	25640	114.3	21.27	C/O	Movebank ID: 468460067
Potos flavus	13	0.02	2018	68.1	63592	331.3	3	H/F	Movebank ID: 468460067
Procyon lotor	6	30	2016	21.2	292.5	48.1	5.52	C/O	Contributed by co-authors
Procyon lotor	2	15	2013	31.4	1335	168.2	5.52	C/O	MovebankID: 11948467
Propithecus verreauxi	28	15	2013	102.7	8789	300.4	3.48	H/F	(Noonan & et al., 2018)
Sus scrofa	26	5	2012	56.9	5675.5	155.8	96.12	H/F	(Noonan & et al., 2018)
Syncerus caffer	6	60	2005	126.6	2284	14.8	580	H/F	(Cross et al., 2016)
Tolypeutes matacus	10	5	2012	4019	4542	61.6	1.2	C/O	Contributed by co-authors
Ursus americanus	17	15	2009	106.6	8608	27.3	99.95	H/F	MovebankID: 8170674
Ursus arctos	12	45	2014	224.3	1420.5	16.8	180.52	H/F	(Noonan & et al., 2018)
Vulpes bengalensis	6	179	2017	174	1429	670.5	2.73	C/O	Movebank ID: 637111097

References

Abrahms, B., 2017. Data from: Suite of simple metrics reveals common movement syndromes across vertebrate taxa. Movebank Data Repository.

Cross, P. C., J. A. Bowers, C. T. Hay, J. Wolhuter, P. Buss, M. Hofmeyr, J. T. du Toit and W. M. Getz, 2016.

Data from: Nonparameteric kernel methods for constructing home ranges and utilization distributions. Movebank Data Repository.

Kays, R. and B. T. Hirsch, 2015. Data from: Stink or swim: techniques to meet the challenges for the study and conservation of small critters that hide, swim or climb and may otherwise make themselves unpleasant. Movebank Data Repository.

McMahon, L. A., J. L. Rachlow, L. A. Shipley and J. S. Forbey, 2017. Data from: Evaluation of micro-GPS receivers for tracking small-bodied mammals. Movebank Data Repository.

Morato, R. G., J. J. Thompson, A. Paviolo, J. A. de La Torre, F. Lima, R. T. McBride Jr, R. C. Paula, L. Cullen Jr, L. Silveira, D. L. Kantek et al., 2018. Jaguar movement database: a gps-based movement dataset of an apex predator in the neotropics. Ecology **99**(7):1691–1691.

Noonan, M. J. and et al., 2018. Data from: A comprehensive analysis of autocorrelation and bias in home range estimation. Dryad Digital Repository.
