# Supplementary material for: Effects of body size on estimation of mammalian area requirements
Source: Conserv Biol. 2020 Jun 18;34(4):1017–28. doi: 10.1111/cobi.13495 (PMC7496598; doi:10.1111/cobi.13495)
Supplement: Supplementary file 2 — Supplementary Material [file COBI-34-1017-s002.pdf]

Supporting Information S2 — Individual tracking dataset summaries

Table S2.1: Summary statistics for each of the individual tracking datasets used in the analyses in the main text. Sampling intervals are reported in minutes, and the sampling duration in days. Consistent refers to whether or not the individual exhibited consistent movement behaviour between the first and second halves of the sampling period (see main text for details).

Species	Individual ID	Country	Consistent	Interval	Start Date	End Date	Duration	Locations
Acinonyx jubatus	Flirty	Botswana	N	0.02	2/9/12	2/17/12	8.1	102143
Acinonyx jubatus	Lisette	South Africa	N	0.02	3/17/12	4/16/12	30.1	107081
Aepyceros melampus	1756	Kenya	Y	20	6/3/11	6/11/12	374	25206
Aepyceros melampus	1757	Kenya	Y	20	6/19/11	5/30/12	345.8	23318
Aepyceros melampus	1758	Kenya	Y	20	6/5/11	9/11/12	463.4	30975
Aepyceros melampus	1759	Kenya	Y	20	6/19/11	10/14/11	117	7741
Aepyceros melampus	1760	Kenya	Y	20	6/18/11	8/3/11	46.1	3193
Aepyceros melampus	1761	Kenya	Y	20	6/4/11	8/29/11	85.5	5673
Aepyceros melampus	1762	Kenya	Y	20	6/5/11	3/7/12	275.9	18505
Aepyceros melampus	1763	Kenya	Y	20	6/19/11	4/22/12	307.8	20217
Aepyceros melampus	1764	Kenya	Y	20	6/24/11	6/7/12	349.2	23358
Aepyceros melampus	1765	Kenya	Y	20	6/5/11	11/8/11	155.7	10361
Aepyceros melampus	1766	Kenya	Y	20	6/26/11	10/25/11	120.8	8103
Aepyceros melampus	1767	Kenya	Y	20	6/19/11	4/3/12	288.9	18881
Aepyceros melampus	1768	Kenya	Y	20	6/1/11	6/16/12	380.3	25516
Aepyceros melampus	1769	Kenya	N	20	6/4/11	8/3/11	59.8	4093
Aepyceros melampus	1770	Kenya	Y	20	6/4/11	8/4/12	426.4	28264
Aepyceros melampus	1771	Kenya	Y	20	6/19/11	5/9/12	325.2	21241
Aepyceros melampus	1772	Kenya	Y	20	6/26/11	9/5/11	71.2	4716
Aepyceros melampus	1773	Kenya	Y	20	6/19/11	4/9/12	295.5	19623
Aepyceros melampus	1774	Kenya	Y	20	6/19/11	5/9/12	325.1	21507
Aepyceros melampus	1775	Kenya	Y	20	6/19/11	5/24/12	340.6	21771
Aepyceros melampus	1776	Kenya	Y	20	5/4/12	5/6/12	1.5	106
Alces alces	11	U.S.A.	Y	90	12/16/04	8/1/06	592.8	9358
Alces alces	117	U.S.A.	Y	60	2/15/05	2/17/07	731.3	11400
Alces alces	13	U.S.A.	Y	90	12/17/04	8/4/06	595.8	7527
Alces alces	14	U.S.A.	Y	90	12/16/04	8/1/06	593	9427
Alces alces	213	U.S.A.	Y	60	2/17/08	5/1/10	803.7	10493
Alces alces	231	U.S.A.	Y	60	2/17/08	4/30/10	803.2	10201
Alces alces	F01	U.S.A.	Y	60	2/24/11	2/12/13	719	17216
Alces alces	F02	U.S.A.	Y	60	2/24/11	2/12/13	718.9	17173
Alces alces	F04	U.S.A.	Y	60	2/23/11	2/11/13	718.8	17074
Alces alces	F09	U.S.A.	Y	60	2/24/11	2/14/13	720.8	17255
Alces alces	F27	U.S.A.	Y	60	2/23/11	2/12/13	719.7	17090
Antidorcas marsupialis	springbok 1	Namibia	Y	60	9/2/09	5/23/10	264	6333
Antidorcas marsupialis	springbok 10	Namibia	Y	60	8/30/09	4/13/10	226.9	5446
Antidorcas marsupialis	springbok 2	Namibia	N	60	8/30/09	11/18/10	446	10590
Antidorcas marsupialis	springbok 5	Namibia	Y	60	9/3/09	4/14/10	224	5376
Antidorcas marsupialis	springbok 6	Namibia	Y	60	9/3/09	4/16/10	225.9	3348
Antidorcas marsupialis	springbok 7	Namibia	Y	60	8/30/09	4/14/10	227.9	5473
Antidorcas marsupialis	springbok 8	Namibia	Y	60	8/30/09	5/23/10	266.9	6407
Antilocapra americana	30	U.S.A.	Y	480	1/27/02	12/16/03	687.5	1212
Antilocapra americana	48	U.S.A.	Y	480	1/26/02	12/16/03	688.5	1213
Antilocapra americana	57	U.S.A.	Y	484	3/31/02	2/11/03	316.7	259
Ateles geoffroyi	Chibi 4693	Panama	Y	0.02	12/11/15	2/20/16	71.3	77038
Ateles geoffroyi	Limon 5215	Panama	Y	0.02	12/1/17	6/14/18	195.1	139034
Ateles geoffroyi	Zola 5212	Panama	Y	0.02	12/13/17	6/1/18	169.9	125302
Beatragus hunteri	11482	Kenya	N	60	8/13/12	4/6/15	966.6	22568
Beatragus hunteri	11483	Kenya	Y	60	12/13/12	4/5/15	843.1	20029
Beatragus hunteri	11486	Kenya	Y	60	12/13/12	4/6/15	844.2	20076
Beatragus hunteri	11487	Kenya	N	60	8/13/12	12/11/12	120.5	2839
Brachylagus idahoensis	CG201515	U.S.A.	Y	15	7/1/15	7/21/15	19.2	1033
Brachylagus idahoensis	CG201516	U.S.A.	Y	15	7/14/15	7/22/15	7.2	392
Brachylagus idahoensis	CG201519	U.S.A.	Y	15	7/1/15	7/9/15	7	308
Brachylagus idahoensis	CG201521	U.S.A.	Y	15	6/29/15	7/2/15	3.1	148
Brachylagus idahoensis	CG201523	U.S.A.	Y	15	6/23/15	7/7/15	13.2	740
Brachylagus idahoensis	CG201526	U.S.A.	Y	15	6/30/15	2/16/16	230.7	622
Brachylagus idahoensis	CG201604	U.S.A.	N	60	1/15/16	2/7/16	23.1	196
Brachylagus idahoensis	CG201606	U.S.A.	N	60	2/18/16	3/1/16	11.9	112
Brachylagus idahoensis	CG201608	U.S.A.	Y	60	1/9/16	2/6/16	27	218
Brachylagus idahoensis	CG201611	U.S.A.	N	60	3/2/16	3/15/16	13	145
Brachylagus idahoensis	CG201612	U.S.A.	Y	60	3/2/16	3/17/16	14.3	166
Canis aureus	Jackal 07	India	Y	15	11/25/16	6/9/17	196.4	5438
Canis aureus	Jackal 09	India	Y	15	11/15/17	9/5/18	293.4	10845
Canis latrans	C01	U.S.A.	N	15	5/4/09	12/1/09	210.2	7192
Canis latrans	C02	U.S.A.	Y	15	5/5/09	12/1/09	210.2	7184
Canis latrans	C04	U.S.A.	Y	15	5/20/09	12/16/09	210.2	9573
Canis latrans	C05	U.S.A.	Y	15	5/21/09	12/17/09	210.3	9641
Canis latrans	C07	U.S.A.	Y	15	5/23/09	1/11/10	232.9	9493
Canis latrans	C08	U.S.A.	Y	15	5/31/09	9/1/09	92.8	8596
Canis latrans	C09	U.S.A.	N	15	6/5/09	1/7/10	215.9	7743
Canis latrans	C10	U.S.A.	Y	15	4/29/10	12/9/10	223.7	10947
Canis latrans	C11	U.S.A.	Y	15	4/30/10	12/9/10	222.7	11481
Canis latrans	C15	U.S.A.	Y	15	5/11/10	12/9/10	211.7	12501
Canis latrans	C16	U.S.A.	Y	15	5/18/10	12/15/10	210.5	11820
Canis latrans	C17	U.S.A.	Y	15	6/3/10	5/20/11	351	11721
Canis latrans	C20	U.S.A.	N	15	6/5/10	11/1/10	148.8	10112
Canis latrans	C23	U.S.A.	Y	15	3/10/11	9/2/11	175.9	11129

Continued on next page

Table S2.1 – continued from previous page

Species	Individual ID	Country	Consistent	Interval	Start Date	End Date	Duration	Locations
Canis latrans	C24	U.S.A.	N	2100	3/14/11	4/12/11	28.6	16
Canis latrans	C25	U.S.A.	Y	15	3/18/11	8/19/11	153.9	10227
Canis latrans	C26	U.S.A.	N	15	5/9/11	9/6/11	120	11205
Canis latrans	C27	U.S.A.	Y	15	5/16/11	10/1/11	137.2	12293
Canis latrans	30808	U.S.A.	Y	180	2/26/11	6/23/11	116.2	950
Canis latrans	30811	U.S.A.	N	180	2/27/11	3/5/12	371.7	1117
Canis latrans	30812	U.S.A.	N	180	2/27/11	6/29/12	488.2	3693
Canis latrans	30818	U.S.A.	Y	180	2/26/11	1/21/12	328.2	2598
Canis latrans	30820	U.S.A.	Y	180	2/27/11	8/5/12	524.2	4136
Canis latrans	30821	U.S.A.	Y	180	2/26/11	1/17/12	324.7	2559
Canis latrans	30822	U.S.A.	Y	180	2/3/11	1/16/12	346.7	2702
Canis latrans	30835	U.S.A.	Y	180	2/26/11	8/5/12	525.3	3753
Canis latrans	30836	U.S.A.	Y	180	3/1/11	12/24/11	297.1	2231
Canis latrans	30837	U.S.A.	Y	180	2/26/11	5/28/12	456.6	3649
Canis latrans	30838	U.S.A.	N	180	2/26/11	11/11/11	257.6	2067
Canis latrans	30839	U.S.A.	N	180	2/27/11	7/1/12	490	3810
Canis latrans	30840	U.S.A.	Y	180	3/1/11	3/3/12	367.3	922
Canis latrans	30841	U.S.A.	Y	180	2/25/11	6/29/12	490	3874
Canis latrans	30842	U.S.A.	Y	180	3/2/11	2/21/12	355.4	2743
Canis latrans	30843	U.S.A.	Y	180	2/24/11	6/28/12	490	3902
Canis latrans	30844	U.S.A.	Y	180	2/25/11	6/7/11	101.6	883
Canis latrans	30845	U.S.A.	Y	180	2/24/11	6/24/12	485.5	3723
Canis latrans	30846	U.S.A.	N	180	2/25/11	8/5/12	526.4	4035
Canis latrans	30847	U.S.A.	N	180	3/1/11	4/5/12	400.4	3096
Canis latrans	30848	U.S.A.	N	180	3/2/11	7/6/12	491.6	3898
Canis latrans	30849	U.S.A.	Y	180	2/26/11	6/15/12	474.5	4771
Canis latrans	30851	U.S.A.	Y	180	3/2/11	7/10/12	495.9	3886
Canis latrans	30852	U.S.A.	Y	180	2/27/11	10/7/11	222.1	1784
Canis latrans	30853	U.S.A.	Y	180	2/26/11	1/29/12	336.6	2266
Canis lupus	W01	U.S.A.	Y	15	5/2/09	11/29/09	210.5	6876
Canis lupus	W02	U.S.A.	Y	15	5/4/09	11/27/09	207	7037
Canis lupus	W05	U.S.A.	Y	15	5/2/10	12/9/10	220.5	12576
Canis lupus	W06	U.S.A.	Y	15	5/4/10	9/14/10	133	12028
Canis lupus	W10	U.S.A.	Y	15	5/21/11	9/6/11	108	9608
Canis lupus familiaris	1	Thailand	N	20	12/25/09	1/7/10	13.8	837
Canis lupus familiaris	2	Thailand	Y	20	12/25/09	1/7/10	13.9	932
Canis lupus familiaris	4	Thailand	N	20	7/10/10	7/22/10	13	790
Canis lupus familiaris	5	Thailand	Y	20	7/10/10	7/22/10	13	778
Canis lupus familiaris	7	Thailand	N	20	7/24/10	8/8/10	16	968
Canis lupus familiaris	9	Thailand	Y	20	8/14/10	9/1/10	19	1255
Canis lupus familiaris	10	Thailand	Y	22	8/14/10	9/1/10	18.9	256
Canis lupus familiaris	14	Thailand	Y	20	9/6/10	9/20/10	15	1016
Canis lupus familiaris	15	Thailand	N	20	10/12/10	11/2/10	22	1456
Canis lupus familiaris	16	Thailand	N	21	10/12/10	11/2/10	21.8	501
Canis lupus familiaris	18	Thailand	Y	20	11/17/10	12/2/10	15.8	967
Canis lupus familiaris	20	Thailand	N	20	11/18/10	12/3/10	15.7	1043
Canis lupus familiaris	21	Thailand	Y	20	1/21/11	2/4/11	14	953
Canis lupus familiaris	22	Thailand	N	20	1/22/11	2/4/11	13.8	958
Canis mesomelas	CM05	Namibia	Y	60	2/7/09	5/2/09	84.8	1873
Canis mesomelas	CM08	Namibia	N	60	2/8/09	6/29/09	141.1	3374
Canis mesomelas	CM09	Namibia	N	60	2/8/09	7/7/10	513.3	11827
Canis mesomelas	CM10	Namibia	Y	60	2/8/09	4/1/09	51.5	1235
Canis mesomelas	CM11	Namibia	N	60	2/9/09	4/16/10	430.8	11508
Canis mesomelas	CM15	Namibia	N	60	2/16/09	6/21/10	490.8	11148
Canis mesomelas	CM18	Namibia	Y	60	2/23/09	4/1/09	37.2	884
Canis mesomelas	CM23	Namibia	N	60	3/1/09	5/3/09	63	1481
Canis mesomelas	CM26	Namibia	N	60	3/3/09	5/2/10	425.3	10700
Canis mesomelas	CM33	Namibia	N	60	3/9/09	11/13/09	248.7	5522
Canis mesomelas	CM36	Namibia	N	60	4/5/09	7/30/10	480.5	3128
Canis mesomelas	CM40	Namibia	Y	10	4/8/09	7/6/09	89.9	7303
Canis mesomelas	CM42	Namibia	Y	20	4/9/09	7/18/09	100.8	2662
Canis mesomelas	CM44	Namibia	N	60	4/14/09	7/18/10	460.5	9914
Canis mesomelas	CM47	Namibia	Y	60	4/22/09	6/24/10	428.8	11358
Canis mesomelas	CM62	Namibia	N	60	7/30/09	10/8/10	436	9532
Canis mesomelas	CM69	Namibia	N	60	12/14/09	1/16/11	398.4	10025
Canis mesomelas	CM72	Namibia	Y	60	12/18/09	3/30/10	102.6	2875
Canis mesomelas	CM83	Namibia	N	60	12/27/09	10/8/10	284.3	8101
Canis mesomelas	CM95	Namibia	Y	60	7/13/10	9/29/10	77.6	1439
Cebus capucinus	Bob 4661	Panama	N	0	12/15/17	3/1/18	76.2	81080
Cebus capucinus	Da Vinci 5764	Panama	N	0	12/15/17	3/30/18	105.1	111292
Cebus capucinus	Ibeth 4654	Panama	N	0	12/25/15	3/31/16	97	103986
Cebus capucinus	Martinelli 5763	Panama	N	0	12/15/17	3/12/18	87.4	93202
Cebus capucinus	Mimi 4660	Panama	N	0	12/15/15	3/12/16	88.3	94886
Cebus capucinus	Norah 4655	Panama	N	0	12/15/17	2/14/18	61.3	66527
Cebus capucinus	Olga 4657	Panama	N	0	12/15/15	2/20/16	67.2	71865
Cebus capucinus	Valoy 5766	Panama	N	0	12/15/17	3/12/18	87.5	91635
Cerdocyon thous	150011	Brazil	Y	5	6/15/12	8/5/12	50.9	13582
Cerdocyon thous	150041	Brazil	N	5	6/17/12	8/10/12	53.9	15379
Cerdocyon thous	150102	Brazil	Y	5	6/14/12	7/23/12	38.2	7132
Cerdocyon thous	150312	Brazil	Y	5	6/14/12	7/4/12	20.4	2300
Cerdocyon thous	150402	Brazil	Y	5	6/13/12	8/12/12	60.3	14876
Cerdocyon thous	150462	Brazil	Y	5	6/15/12	8/16/12	62.2	12968
Cerdocyon thous	150552	Brazil	N	5	6/15/12	7/24/12	38.4	8399
Cerdocyon thous	150681	Brazil	Y	6	6/17/12	7/10/12	23.1	2918
Cerdocyon thous	163181	Brazil	N	0	1/1/13	2/28/13	57.6	22991
Cerdocyon thous	164820	Brazil	Y	5	6/14/12	7/7/12	22.5	5126
Cerdocyon thous	164886	Brazil	Y	5	2/20/13	4/4/13	43.1	8111
Cerdocyon thous	164900	Brazil	Y	5	6/17/12	8/3/12	46.7	12690

Continued on next page

Table S2.1 – continued from previous page

Species	Individual ID	Country	Consistent	Interval	Start Date	End Date	Duration	Locations
Cerdocyon thous	164957	Brazil	Y	5	5/28/13	6/16/13	19.7	1811
Cerdocyon thous	1649671	Brazil	N	5	4/12/13	5/23/13	41.1	8160
Cerdocyon thous	165164	Brazil	Y	5	2/25/13	4/2/13	36.3	5608
Cerdocyon thous	165194	Brazil	Y	5	5/30/13	7/5/13	36.1	6746
Cerdocyon thous	1651941	Brazil	Y	4	4/14/13	6/17/13	64.2	19844
Cerdocyon thous	165224	Brazil	Y	4	2/20/13	3/16/13	24.3	6642
Cerdocyon thous	165252	Brazil	Y	5	4/14/13	5/25/13	41	10005
Cerdocyon thous	1652521	Brazil	Y	5	5/27/13	7/14/13	48.1	11020
Cervus canadensis	17	U.S.A.	Y	180	2/19/00	2/23/02	734.9	3640
Cervus canadensis	197	U.S.A.	Y	60	1/28/10	2/15/12	747.8	17945
Cervus canadensis	20	U.S.A.	Y	180	2/20/00	2/23/02	734.5	3728
Cervus canadensis	205	U.S.A.	Y	60	1/28/10	1/22/12	724.1	17377
Cervus canadensis	216	U.S.A.	Y	60	1/28/10	2/17/12	750	17999
Cervus canadensis	219	U.S.A.	Y	60	1/28/10	1/23/12	724.9	17397
Cervus canadensis	228	U.S.A.	Y	60	1/28/10	1/22/12	724.2	17379
Cervus canadensis	28	U.S.A.	Y	180	2/19/00	2/23/02	734.9	3748
Cervus canadensis	34	U.S.A.	Y	180	1/24/07	3/31/10	1161.6	6429
Cervus canadensis	37	U.S.A.	Y	180	1/24/07	3/31/10	1161.9	6400
Chlorocebus pygerythrus	Cp BR GG	Kenya	Y	15	1/17/14	1/30/15	377.9	35597
Chlorocebus pygerythrus	Cp BR TZ	Kenya	Y	15	1/17/14	1/14/15	361.9	34037
Chlorocebus pygerythrus	Cp CT AS	Kenya	N	15	3/23/14	9/16/14	177.2	16773
Chlorocebus pygerythrus	Cp CT CO	Kenya	N	15	3/26/14	1/14/15	294.4	27410
Chlorocebus pygerythrus	Cp CT GS	Kenya	Y	15	1/16/14	2/24/14	38.4	3652
Chlorocebus pygerythrus	Cp FG BU	Kenya	N	15	1/21/14	1/30/15	373.9	35402
Chlorocebus pygerythrus	Cp HP CD	Kenya	Y	15	1/16/14	3/2/14	45	4296
Chlorocebus pygerythrus	Cp HP CV	Kenya	Y	15	3/24/14	5/31/14	67.9	6470
Chlorocebus pygerythrus	Cp HP PI	Kenya	Y	15	1/16/14	1/9/15	357.9	33668
Chlorocebus pygerythrus	Cp KU ME	Kenya	Y	15	1/20/14	1/21/15	366	34414
Chlorocebus pygerythrus	Cp KU MK	Kenya	Y	15	1/20/14	12/15/14	329.2	31406
Chrysocyon brachyurus	Amadeo	Brazil	Y	121	3/20/07	10/20/08	579.8	3514
Chrysocyon brachyurus	Bolt	Brazil	Y	60	10/1/13	7/21/14	293.2	7005
Chrysocyon brachyurus	Gamba	Brazil	Y	240	4/30/09	8/5/09	96.7	562
Chrysocyon brachyurus	Henry	Brazil	Y	240	5/12/10	1/22/11	254.5	1451
Chrysocyon brachyurus	Jurema	Brazil	Y	240	9/3/09	1/25/11	508.7	2846
Chrysocyon brachyurus	Lais	Brazil	Y	120	5/3/07	7/28/08	452	3726
Chrysocyon brachyurus	Loba	Brazil	Y	241	6/15/12	12/29/13	562.8	2317
Chrysocyon brachyurus	Luna	Brazil	Y	60	2/28/13	12/19/13	293.3	5775
Chrysocyon brachyurus	Miro	Brazil	Y	240	3/25/11	4/9/12	381.2	2565
Chrysocyon brachyurus	Nilde	Brazil	Y	120	3/30/11	5/27/11	58.2	618
Chrysocyon brachyurus	Rose	Brazil	Y	60	7/20/14	9/17/14	59	1427
Chrysocyon brachyurus	Samurai	Brazil	Y	240	8/26/09	11/19/09	84.7	496
Chrysocyon brachyurus	Tay	Brazil	Y	120	3/14/07	12/5/09	996.4	8760
Connochaetes taurinus	Fifteen	Kenya	Y	60	5/30/10	12/10/12	925.3	11659
Connochaetes taurinus	Karbolo	Kenya	Y	60	10/19/10	12/8/11	415.3	6152
Connochaetes taurinus	Kayioni	Kenya	N	60	5/26/10	6/13/12	749.6	11461
Connochaetes taurinus	Kikaya	Kenya	N	60	10/21/10	10/17/12	727.4	10799
Connochaetes taurinus	Kiranto	Kenya	N	60	10/17/10	1/15/13	821	12758
Connochaetes taurinus	Koyiaki	Kenya	N	60	5/28/10	3/18/11	294.5	4444
Connochaetes taurinus	Laingoni	Kenya	Y	60	10/15/10	3/17/12	518.9	7889
Connochaetes taurinus	Ledama	Kenya	N	60	5/26/10	4/7/12	682.2	8434
Connochaetes taurinus	Lempaash	Kenya	Y	60	10/11/10	7/8/11	270	4218
Connochaetes taurinus	Lengaruani	Kenya	Y	60	10/11/10	6/10/12	608	8611
Connochaetes taurinus	Motorogi	Kenya	N	60	5/26/10	9/13/11	474.1	7460
Connochaetes taurinus	Nagol	Kenya	Y	60	5/27/10	1/15/13	963.9	14863
Connochaetes taurinus	Naiba	Kenya	N	60	5/28/10	8/18/10	81.8	1338
Connochaetes taurinus	Naserian	Kenya	N	60	5/27/10	1/15/13	964.1	15039
Connochaetes taurinus	Ne Mpaash	Kenya	N	60	10/16/10	4/10/12	541.5	7665
Connochaetes taurinus	Nentepesi	Kenya	Y	60	10/10/10	1/15/13	827.9	12926
Connochaetes taurinus	Nesimei	Kenya	Y	60	10/10/10	12/29/12	811	8207
Connochaetes taurinus	Nkoko	Kenya	N	60	5/27/10	3/28/11	305.4	4718
Connochaetes taurinus	Noontare	Kenya	Y	60	10/16/10	1/15/13	822	11877
Connochaetes taurinus	Ntishya	Kenya	Y	60	10/19/10	11/20/12	763.2	12008
Connochaetes taurinus	Ole Nagol	Kenya	Y	60	10/16/10	12/25/10	69.3	1123
Connochaetes taurinus	Olope	Kenya	Y	60	5/25/10	8/16/11	447.9	6943
Connochaetes taurinus	Paita	Kenya	N	60	10/18/10	1/15/13	819.7	12470
Connochaetes taurinus	Peria	Kenya	N	60	10/19/10	1/11/13	814.7	12560
Connochaetes taurinus	Reiyo	Kenya	N	60	10/12/10	1/14/13	825.4	12657
Connochaetes taurinus	Sawani	Kenya	Y	60	10/20/10	6/14/11	236.3	3663
Connochaetes taurinus	Sotua	Kenya	Y	60	10/15/10	9/13/11	332.7	4864
Connochaetes taurinus	Taletian	Kenya	N	60	5/29/10	6/14/10	16.4	244
Cuon alpinus	Anucha_KARNWS	Thailand	Y	479	2/25/15	8/5/15	160.3	380
Cuon alpinus	FARN_KYNP	Thailand	N	362	2/2/17	7/17/17	165.7	254
Cuon alpinus	Pakchi_KYNP	Thailand	Y	361	2/8/17	4/22/17	72.8	179
Cuon alpinus	Valentine_SLP	Thailand	Y	240	2/15/15	11/17/15	275	880
Dasyprocta punctata	Lab Rat	Panama	Y	15	1/12/09	1/20/09	7.6	214
Didelphis virginiana	Hilda	U.S.A.	Y	15	9/5/13	10/23/13	48.3	242
Elephas maximus maximus	Asokamala	Sri Lanka	Y	240	6/6/08	7/19/08	43.2	146
Elephas maximus maximus	Biso	Sri Lanka	Y	480	11/24/17	3/24/18	120.3	199
Elephas maximus maximus	BisuMenike	Sri Lanka	Y	240	12/17/04	11/2/05	320	1289
Elephas maximus maximus	Chandi	Sri Lanka	Y	480	3/18/09	6/12/09	86	157
Elephas maximus maximus	Chintha	Sri Lanka	Y	240	6/18/17	7/1/18	378	2259
Elephas maximus maximus	Dase	Sri Lanka	Y	60	11/11/10	9/8/11	301.3	5710
Elephas maximus maximus	Devi	Sri Lanka	Y	240	4/28/18	12/13/18	229.2	1359
Elephas maximus maximus	Dexie	Sri Lanka	Y	60	10/12/13	8/16/16	1039.9	13087
Elephas maximus maximus	Disala	Sri Lanka	N	480	10/7/06	3/14/09	889	2212
Elephas maximus maximus	Dushya	Sri Lanka	Y	240	10/6/15	5/23/18	960.8	2010
Elephas maximus maximus	Ekes	Sri Lanka	N	480	1/13/09	10/18/11	1008.7	3007
Elephas maximus maximus	Galli	Sri Lanka	Y	240	9/9/08	9/19/09	375	1749

Continued on next page

Table S2.1 – continued from previous page

Species	Individual ID	Country	Consistent	Interval	Start Date	End Date	Duration	Locations
Elephas maximus maximus	Himali	Sri Lanka	Y	240	10/5/15	1/25/16	112.7	592
Elephas maximus maximus	Homey	Sri Lanka	Y	240	3/25/06	8/3/07	496.8	2096
Elephas maximus maximus	Hura	Sri Lanka	Y	60	11/7/10	7/9/13	975.4	15907
Elephas maximus maximus	Hurululiya	Sri Lanka	Y	240	11/6/09	7/20/12	987.3	4394
Elephas maximus maximus	Induwara	Sri Lanka	N	240	6/23/17	2/15/18	237.7	1422
Elephas maximus maximus	Jambu	Sri Lanka	N	240	10/17/13	4/13/14	178	1043
Elephas maximus maximus	Janaki	Sri Lanka	Y	240	9/13/13	3/30/16	929.8	5526
Elephas maximus maximus	Kala	Sri Lanka	Y	240	11/8/09	4/16/12	890.7	5090
Elephas maximus maximus	Kandula	Sri Lanka	N	240	5/9/05	2/24/08	1021	4598
Elephas maximus maximus	Karattaya	Sri Lanka	Y	240	11/6/06	8/2/07	269	1543
Elephas maximus maximus	Kavan	Sri Lanka	Y	240	10/31/04	9/2/05	306.7	1699
Elephas maximus maximus	Kumari	Sri Lanka	Y	960	9/11/09	6/12/12	1005.3	1249
Elephas maximus maximus	Kuveni	Sri Lanka	Y	240	5/3/18	5/21/19	383	2242
Elephas maximus maximus	Madara	Sri Lanka	Y	240	9/8/09	7/7/11	667.7	3933
Elephas maximus maximus	Madhumali	Sri Lanka	Y	240	9/12/13	2/15/14	156.5	937
Elephas maximus maximus	Mihiri	Sri Lanka	Y	240	11/6/09	8/11/12	1008.8	4708
Elephas maximus maximus	Najaah	Sri Lanka	Y	240	10/16/13	8/28/17	1412.2	8387
Elephas maximus maximus	Nancy	Sri Lanka	Y	240	11/9/09	4/27/12	900.7	2944
Elephas maximus maximus	Nandimithra	Sri Lanka	Y	240	7/1/17	10/8/17	99	595
Elephas maximus maximus	Parakum	Sri Lanka	N	240	11/6/09	12/9/09	33.2	171
Elephas maximus maximus	Ranmali	Sri Lanka	Y	240	7/6/17	2/24/18	233.7	1397
Elephas maximus maximus	Rolli	Sri Lanka	Y	240	8/16/17	10/1/18	411	2060
Elephas maximus maximus	Sakunthala	Sri Lanka	Y	240	9/19/09	1/8/12	841.7	4922
Elephas maximus maximus	Samare	Sri Lanka	Y	240	7/30/12	6/24/14	694.7	2974
Elephas maximus maximus	Samudra	Sri Lanka	Y	240	11/11/09	10/23/11	711.2	3227
Elephas maximus maximus	Sapumali	Sri Lanka	Y	360	6/20/09	3/8/10	261.4	867
Elephas maximus maximus	Sita	Sri Lanka	Y	240	9/26/08	3/8/09	163	708
Elephas maximus maximus	Soma	Sri Lanka	N	240	11/10/09	2/14/12	826.3	3690
Elephas maximus maximus	Sugala	Sri Lanka	Y	240	9/3/09	7/1/12	1032.5	5117
Elephas maximus maximus	TaraDevi	Sri Lanka	Y	240	11/23/17	5/21/18	179	1072
Elephas maximus maximus	TekkaNanda	Sri Lanka	Y	240	6/5/08	11/20/09	533	2544
Elephas maximus maximus	Thaga	Sri Lanka	Y	240	9/17/09	3/31/10	195.7	1172
Elephas maximus maximus	Thushari	Sri Lanka	Y	240	9/9/09	6/17/11	646.9	3634
Elephas maximus maximus	Udani	Sri Lanka	Y	240	9/2/09	8/1/12	1064.3	5409
Elephas maximus maximus	Uma	Sri Lanka	Y	240	9/18/09	5/6/13	1326.3	7619
Elephas maximus maximus	Valli	Sri Lanka	N	240	7/11/09	1/15/13	1284.7	7648
Elephas maximus maximus	Vasana	Sri Lanka	Y	1680	7/28/12	3/20/13	235	83
Elephas maximus maximus	Wanamali	Sri Lanka	Y	240	9/17/09	11/11/11	785.5	3924
Elephas maximus maximus	Wasaba	Sri Lanka	Y	240	2/3/07	2/6/08	368.8	1149
Elephas maximus maximus	Wira	Sri Lanka	Y	240	9/11/09	8/15/11	702.7	3854
Elephas maximus sumatranus	Anna	Indonesia	Y	120	7/24/12	12/8/15	1231.1	10215
Elephas maximus sumatranus	Bella	Indonesia	Y	240	7/28/12	6/16/13	322.3	1912
Elephas maximus sumatranus	Cinta	Indonesia	Y	120	7/29/12	12/8/15	1226.1	10252
Elephas maximus sumatranus	Dadang	Indonesia	Y	120	7/31/12	12/7/15	1224.6	8095
Elephas maximus sumatranus	Elena	Indonesia	Y	240	8/1/12	12/28/13	514.3	570
Elephas maximus sumatranus	Freda	Indonesia	Y	120	7/29/13	12/8/15	861.2	9039
Elephas maximus sumatranus	Haris	Indonesia	Y	120	11/16/14	12/7/15	386	4268
Elephas maximus sumatranus	Indah	Indonesia	Y	120	1/9/15	12/6/15	331.4	3522
Equus hemionus hemionus	2	Mongolia	Y	13	7/22/09	7/20/10	362.8	24972
Equus hemionus hemionus	3	Mongolia	Y	13	7/20/09	7/20/10	364.8	40638
Equus hemionus hemionus	4	Mongolia	Y	13	7/20/09	7/20/10	364.8	40203
Equus hemionus hemionus	5	Mongolia	Y	12	7/23/09	7/20/10	361.8	31227
Equus hemionus hemionus	6	Mongolia	Y	13	7/20/09	7/20/10	364.9	14179
Equus hemionus hemionus	620	Mongolia	Y	15	7/5/07	6/20/08	350.6	33016
Equus hemionus hemionus	6441	Mongolia	Y	15	7/22/09	7/20/10	362.8	34811
Equus hemionus hemionus	6446	Mongolia	Y	15	7/21/09	7/20/10	363.7	34904
Equus hemionus hemionus	7	Mongolia	Y	14	7/24/09	7/20/10	360.7	37383
Equus hemionus hemionus	7376	Mongolia	Y	15	7/23/09	7/20/10	361.8	34669
Equus quagga	zebra 4	Namibia	Y	60	4/20/09	4/30/10	374.5	8598
Equus quagga	zebra 5	Namibia	Y	60	4/20/09	8/29/10	496	10942
Equus quagga	zebra 6	Namibia	Y	60	10/6/09	8/29/10	327	7849
Equus quagga	zebra 7	Namibia	Y	60	10/6/09	9/1/10	330	7791
Equus quagga	zebra 8	Namibia	Y	60	10/6/09	8/29/10	327	7848
Equus quagga	zebra 9	Namibia	Y	60	10/6/09	8/29/10	327	7849
Erinaceus europaeus	Black_EE_Male	U.K.	Y	20	5/15/14	6/11/14	26.2	91
Erinaceus europaeus	Blue_G_Male	U.K.	Y	20	6/7/13	8/15/13	69.1	207
Erinaceus europaeus	Green_G_Female	U.K.	Y	20	5/22/13	5/30/13	7	130
Erinaceus europaeus	Green_I_Male	U.K.	Y	20	10/1/13	5/14/14	225	143
Erinaceus europaeus	Grey_B_Female	U.K.	Y	20	6/6/13	6/13/13	7	113
Erinaceus europaeus	Grey_H_Female	U.K.	Y	20	6/28/13	6/17/14	354	407
Erinaceus europaeus	Orange_E_Female	U.K.	Y	20	8/9/13	6/22/14	317.9	254
Erinaceus europaeus	Purple_D_Female	U.K.	Y	20	5/1/14	5/4/14	2.1	46
Erinaceus europaeus	Red_D_Male	U.K.	Y	20	6/27/13	6/24/14	361	182
Erinaceus europaeus	Red_I_Female	U.K.	N	20	8/8/13	10/7/13	59.3	301
Erinaceus europaeus	Red_II_Male	U.K.	Y	20	5/30/14	6/15/14	15.2	132
Erinaceus europaeus	White_H_Male	U.K.	Y	20	6/20/13	6/27/13	6.3	110
Erinaceus europaeus	Yellow_F_Male	U.K.	Y	20	6/6/13	6/13/13	6.1	97
Erinaceus europaeus	Yellow_H_Male	U.K.	Y	20	7/18/13	8/6/13	18.2	186
Eulemur rufifrons	groupA	Madagascar	Y	30	6/23/09	9/8/09	76.9	3506
Eulemur rufifrons	groupB	Madagascar	Y	5	7/29/09	8/24/09	26.3	6705
Eulemur rufifrons	groupF	Madagascar	Y	30	6/24/09	9/8/09	76	3549
Eulemur rufifrons	groupJ	Madagascar	N	30	1/13/09	5/16/09	122.4	4488
Euphractus sexcinctus	ES01	Brazil	Y	5	3/28/14	4/1/14	4.2	169
Euphractus sexcinctus	ES02	Brazil	Y	5	3/8/14	7/17/14	131.4	506
Euphractus sexcinctus	ES06	Brazil	Y	5	3/15/14	4/5/14	21.2	1331
Euphractus sexcinctus	ES07	Brazil	N	5	4/22/14	5/6/14	14.1	944
Euphractus sexcinctus	ES10	Brazil	Y	5	4/20/14	4/24/14	4.3	264
Euphractus sexcinctus	ES11	Brazil	Y	5	3/20/14	4/1/14	12.4	340

Continued on next page

Table S2.1 – continued from previous page

Species	Individual ID	Country	Consistent	Interval	Start Date	End Date	Duration	Locations
Euphractus sexcinctus	ES12	Brazil	Y	5	4/29/14	5/15/14	16.4	1141
Euphractus sexcinctus	ES13	Brazil	Y	5	4/19/14	4/25/14	6.1	195
Euphractus sexcinctus	ES14	Brazil	N	5	4/22/14	4/23/14	1.4	59
Felis silvestris	WK1	Germany	Y	15	6/1/14	9/15/14	106.2	2006
Felis silvestris	WK2	Netherlands	Y	120	10/15/14	3/13/15	148.4	742
Felis silvestris	WK3	Netherlands	Y	240	10/30/14	12/5/14	35.8	128
Felis silvestris	WK4	Netherlands	Y	120	12/14/14	3/7/15	82.6	391
Felis silvestris	WK5	Netherlands	Y	120	2/12/15	3/31/15	46.7	275
Giraffa camelopardalis reticulata	st2010-2717	Kenya	Y	60	6/11/17	1/23/19	591.1	14150
Giraffa camelopardalis reticulata	st2010-2719	Kenya	N	60	6/11/17	1/23/19	591.1	12768
Giraffa camelopardalis reticulata	st2010-2721	Kenya	Y	60	6/11/17	9/20/17	101.1	2428
Giraffa camelopardalis reticulata	st2010-2723	Kenya	Y	60	6/11/17	8/20/18	435.1	10388
Giraffa camelopardalis reticulata	st2010-2724	Kenya	Y	60	6/11/17	7/24/17	43.9	1054
Giraffa camelopardalis reticulata	st2010-2726	Kenya	Y	60	6/11/17	1/26/18	229.1	5476
Giraffa camelopardalis reticulata	st2010-2732	Kenya	Y	60	6/11/17	10/6/17	117.6	2823
Hyaena brunnea	Alfred	South Africa	N	60	9/30/09	5/12/10	224.8	1047
Hyaena brunnea	Bonnie	South Africa	Y	156	8/5/08	9/16/08	41.9	244
Hyaena brunnea	Bryan	South Africa	Y	365	4/21/07	6/5/07	45	102
Hyaena brunnea	Carol	South Africa	Y	1080	11/24/09	4/22/11	513.3	728
Hyaena brunnea	Lucky	South Africa	Y	299	9/27/09	7/27/10	303.2	755
Hyaena brunnea	Lucy	South Africa	N	121	4/13/10	10/26/10	196	943
Hyaena brunnea	Mac	South Africa	Y	122	3/9/11	4/4/11	25.3	87
Hyaena brunnea	Theo	South Africa	Y	90	2/9/10	3/17/10	35.2	189
Hyaena brunnea	Tina	South Africa	N	1141	4/10/08	7/23/08	103.7	88
Leopardus pardalis	Barrote	Panama	Y	28	12/5/02	9/29/03	298.1	103
Leopardus pardalis	Bobby	Panama	Y	20	11/7/02	1/13/04	432.1	377
Leopardus pardalis	Encito	Panama	Y	50	5/10/03	12/31/03	234.5	500
Leopardus pardalis	Estrella	Panama	Y	22	2/3/04	6/21/04	138.9	361
Leopardus pardalis	Franja	Panama	Y	176	10/11/02	1/13/04	458.8	287
Leopardus pardalis	Isaac	Panama	Y	25	10/10/02	11/10/03	395.1	271
Leopardus pardalis	Mancha	Panama	N	73	1/14/03	9/12/03	241	198
Leopardus pardalis	Yara	Panama	Y	16	8/15/02	12/31/03	502.5	183
Lepus europaeus	Anastasia	Germany	Y	60	7/13/14	1/15/15	185.8	2674
Lepus europaeus	Christl	Germany	Y	60	7/15/14	12/24/14	161.8	4426
Lepus europaeus	Fips	Germany	Y	60	7/12/14	2/7/15	209.7	3041
Lepus europaeus	Frank	Germany	Y	60	5/12/14	12/2/14	203.6	2999
Lepus europaeus	Goliath	Germany	Y	60	6/27/14	7/3/14	5.6	84
Lepus europaeus	Joko	Germany	Y	60	6/4/14	12/26/14	204.9	2762
Lepus europaeus	Lissi	Germany	N	390	6/3/14	6/21/14	17.7	43
Lepus europaeus	Ludwig	Germany	N	126	7/11/14	7/30/14	19.1	43
Lepus europaeus	Mary	Germany	Y	60	5/14/14	12/15/14	214.7	3196
Lepus europaeus	Matz	Germany	Y	60	7/10/14	2/2/15	207.1	3155
Lepus europaeus	Sophia	Germany	N	60	5/14/14	11/21/14	190.5	2853
Lepus europaeus	Wiwi	Germany	N	60	5/13/14	12/1/14	202	2794
Lepus timidus	150.032	U.K.	N	1	11/20/09	11/23/09	2.6	2594
Loxodonta africana	elephant 1	Namibia	N	60	10/30/08	2/7/10	465.5	8550
Loxodonta africana	elephant 2	Namibia	Y	60	10/30/08	6/23/09	236	5666
Loxodonta africana	elephant 3	Namibia	Y	60	10/30/08	7/9/10	618	14832
Loxodonta africana	elephant 4	Namibia	Y	60	10/30/08	1/2/10	429.8	6652
Loxodonta africana	elephant 5	Namibia	Y	60	10/30/08	8/29/10	668.4	15389
Loxodonta africana	elephant 6	Namibia	Y	60	10/30/08	8/29/10	668.5	15957
Loxodonta africana	elephant 7	Namibia	Y	60	10/30/08	1/14/10	441.4	10578
Loxodonta africana	elephant 8	Namibia	Y	60	10/30/08	8/29/10	668.4	15959
Lynx rufus	BOBCAT_39726	U.S.A.	Y	300	12/12/18	3/10/19	87.6	362
Madoqua guentheri	1145.1	Kenya	Y	10	6/18/10	7/13/10	24.9	2306
Madoqua guentheri	1145.11	Kenya	Y	10	9/7/11	9/16/11	9	914
Madoqua guentheri	1147.11	Kenya	N	10	9/6/11	10/2/11	26	2194
Madoqua guentheri	1148.11	Kenya	Y	10	9/6/11	10/2/11	26.1	2249
Madoqua guentheri	1149.11	Kenya	Y	10	9/6/11	10/2/11	26.1	2160
Madoqua guentheri	1150.11	Kenya	Y	10	9/7/11	10/2/11	25.2	2098
Madoqua guentheri	1150_2.10	Kenya	Y	10	6/18/10	7/13/10	24.8	2413
Madoqua guentheri	1151.11	Kenya	Y	10	9/6/11	10/2/11	26.1	2025
Madoqua guentheri	1152.1	Kenya	N	10	6/18/10	7/13/10	25	2361
Madoqua guentheri	1153.1	Kenya	Y	10	6/18/10	7/13/10	24.9	2417
Madoqua guentheri	1154.1	Kenya	Y	10	6/18/10	7/13/10	24.9	2410
Madoqua guentheri	1155.1	Kenya	N	10	6/18/10	7/8/10	19.9	1684
Madoqua guentheri	1155.11	Kenya	Y	10	9/7/11	9/27/11	20.5	1160
Madoqua guentheri	1156.1	Kenya	Y	10	6/18/10	7/13/10	24.8	2272
Madoqua guentheri	1156.11	Kenya	Y	10	9/6/11	10/2/11	26.1	2190
Martes pennanti	Bob	U.S.A.	N	10	3/8/11	3/18/11	10.2	746
Martes pennanti	Buster	U.S.A.	Y	10	1/25/10	2/7/10	12.9	446
Martes pennanti	Eva	U.S.A.	Y	10	8/16/09	9/13/09	28.3	874
Martes pennanti	Gary	U.S.A.	Y	2	12/22/10	1/22/11	31.1	5966
Martes pennanti	Isabella	U.S.A.	N	10	3/12/11	3/28/11	16.1	686
Martes pennanti	Leroy	U.S.A.	Y	15	2/11/09	3/4/09	20.9	919
Martes pennanti	Lucile	U.S.A.	Y	10	2/11/11	3/3/11	19.4	1349
Martes pennanti	Lupe	U.S.A.	Y	2	12/16/10	1/4/11	18.6	3004
Martes pennanti	Maurice	U.S.A.	Y	10	2/10/11	3/7/11	24.7	1638
Martes pennanti	Phineas	U.S.A.	Y	2	1/19/11	2/11/11	22.4	2436
Martes pennanti	Potato Head	U.S.A.	Y	2	3/18/11	5/28/11	71.4	13925
Martes pennanti	Price	U.S.A.	N	10	12/4/09	12/29/09	24.3	1076
Martes pennanti	Ricky T	U.S.A.	Y	2	2/9/10	3/31/10	49.8	8929
Martes pennanti	Zissou	U.S.A.	Y	2	1/21/11	2/7/11	16.3	1501
Nasua narica	Atlas 4673	Panama	Y	4	1/12/16	2/16/16	35.4	3699
Nasua narica	Avery 4671	Panama	Y	0.02	12/15/17	3/27/18	102.3	114047
Nasua narica	Carlsberg 4673	Panama	Y	0.02	12/15/17	3/23/18	98.1	108675
Nasua narica	Clementina 4672	Panama	Y	0.02	12/14/15	3/18/16	95	92918
Nasua narica	Ellie 4668	Panama	N	0.02	12/14/15	3/3/16	80.5	80575

Continued on next page

Table S2.1 – continued from previous page

Species	Individual ID	Country	Consistent	Interval	Start Date	End Date	Duration	Locations
Nasua narica	Fonta Flora 4689	Panama	Y	0.02	12/15/17	3/26/18	101.4	112891
Nasua narica	Aleja	Panama	Y	15	3/15/10	4/24/10	39.9	1122
Nasua narica	Anna	Panama	Y	21	3/22/10	4/6/10	15	79
Odocoileus hemionus	37	U.S.A.	N	180	1/28/11	3/31/13	792.9	5196
Odocoileus virginianus ATS	WTD_39772	U.S.A.	Y	60	11/29/18	3/12/19	102.8	2460
Odocoileus virginianus ATS	WTD_39775	U.S.A.	N	60	2/24/18	3/12/19	380.6	9117
Odocoileus virginianus ATS	WTD_39776	U.S.A.	N	60	2/18/18	3/12/19	386.8	9265
Odocoileus virginianus ATS	WTD_39777	U.S.A.	Y	60	1/14/18	1/14/19	364.7	8737
Odocoileus virginianus ATS	WTD_39793	U.S.A.	Y	60	1/28/18	3/11/19	407.1	9743
Odocoileus virginianus CTT	WTD_57452	U.S.A.	Y	102	11/14/17	6/15/18	213	2880
Odocoileus virginianus CTT	WTD_59201	U.S.A.	Y	62	12/8/17	6/15/18	188.9	2940
Odocoileus virginianus CTT	WTD_60613	U.S.A.	Y	61	1/15/18	6/15/18	151.4	3136
Odocoileus virginianus CTT	WTD_60860	U.S.A.	N	62	12/10/17	7/19/18	221.1	4226
Odocoileus virginianus CTT	WTD_75363	U.S.A.	Y	60	12/5/18	3/14/19	99.9	2477
Odocoileus virginianus CTT	WTD_76916	U.S.A.	Y	60	1/25/19	3/14/19	48.9	1208
Oryx dammah	52	Chad	Y	120	8/2/17	2/22/19	568.5	7377
Oryx dammah	53	Chad	N	60	7/30/17	11/25/18	482.9	11323
Oryx dammah	54	Chad	Y	239	8/3/17	2/22/19	568.1	4829
Oryx dammah	55	Chad	N	239	7/30/17	2/21/19	570.8	4923
Oryx dammah	56	Chad	Y	120	8/13/16	2/22/19	923.5	11112
Oryx dammah	57	Chad	Y	240	1/21/17	2/18/18	392.9	2131
Oryx dammah	58	Chad	Y	120	1/21/17	2/22/19	762.4	9142
Oryx dammah	61	Chad	Y	60	8/3/17	2/21/19	567.6	8085
Oryx dammah	62	Chad	Y	60	8/3/17	11/23/17	112.2	1636
Oryx dammah	64	Chad	N	60	8/2/17	2/22/19	568.4	13623
Oryx dammah	65	Chad	Y	60	8/2/17	2/22/19	568.5	13623
Oryx dammah	66	Chad	Y	60	8/2/17	1/21/18	171.3	4097
Oryx dammah	67	Chad	N	60	8/2/17	2/21/19	567.5	7255
Oryx dammah	68	Chad	Y	60	8/2/17	2/21/19	567.2	13528
Oryx dammah	70	Chad	N	60	8/2/17	10/21/17	79.2	1897
Oryx dammah	72	Chad	N	240	8/2/17	11/22/18	476.6	4004
Oryx dammah	73	Chad	Y	60	1/21/17	9/10/18	597.3	14307
Oryx dammah	77	Chad	N	60	8/2/17	12/10/18	495	6907
Oryx dammah	78	Chad	N	60	8/2/17	2/22/19	568.7	13616
Oryx dammah	79	Chad	Y	60	8/2/17	2/22/19	568.7	5992
Oryx dammah	80	Chad	Y	240	8/2/17	9/6/18	399.5	3369
Oryx dammah	81	Chad	Y	60	8/2/17	2/21/19	567.7	13572
Oryx dammah	82	Chad	N	120	8/2/17	2/22/19	568.5	7387
Oryx dammah	83	Chad	Y	60	1/21/17	6/18/18	514	12316
Oryx dammah	86	Chad	Y	60	8/2/17	9/25/17	53.3	1187
Oryx dammah	87	Chad	Y	60	7/30/17	9/2/17	34	814
Oryx dammah	88	Chad	Y	60	8/2/17	2/22/19	568.5	10503
Oryx dammah	89	Chad	Y	60	7/30/17	2/22/19	571.2	13690
Oryx dammah	92	Chad	N	60	1/21/17	11/26/18	674	16119
Oryx dammah	94	Chad	Y	60	8/13/16	4/28/18	623.5	14923
Oryx dammah	95	Chad	N	60	8/13/16	5/1/18	626.1	14969
Oryx dammah	96	Chad	N	60	1/21/17	10/23/18	640.8	15326
Oryx dammah	97	Chad	Y	60	8/13/16	10/18/17	431.7	9848
Oryx dammah	99	Chad	N	60	8/13/16	5/2/18	627.3	15012
Oryx dammah	100	Chad	Y	60	8/13/16	5/2/18	627.3	13572
Oryx dammah	101	Chad	N	60	1/21/17	11/27/18	675.1	16153
Oryx dammah	102	Chad	Y	60	8/13/16	1/4/18	509	12164
Oryx dammah	103	Chad	Y	60	8/2/17	2/22/19	568.3	13601
Oryx dammah	105	Chad	N	60	8/13/16	5/12/18	637.7	15061
Oryx dammah	106	Chad	Y	60	8/13/16	1/9/18	513.4	12288
Oryx dammah	108	Chad	N	60	8/13/16	5/1/18	626.4	14996
Oryx dammah	109	Chad	Y	60	8/12/16	11/26/18	835.2	13527
Oryx dammah	111	Chad	N	60	8/12/16	5/2/18	627.4	14998
Oryx dammah	113	Chad	Y	60	8/13/16	8/14/17	366.5	8769
Oryx dammah	114	Chad	N	60	8/13/16	5/1/18	626.9	15018
Oryx dammah	116	Chad	N	60	8/2/17	11/24/18	478.8	11219
Oryx dammah	117	Chad	Y	60	8/2/17	12/21/18	506	9668
Oryx dammah	118	Chad	Y	60	8/13/16	5/7/18	632.1	15001
Oryx dammah	119	Chad	Y	60	8/2/17	7/2/18	333.2	7975
Ovis canadensis	OR812	U.S.A.	N	420	12/5/04	10/2/07	1031.4	2666
Ovis canadensis	OR813	U.S.A.	Y	420	12/4/04	10/1/07	1030.7	2851
Ovis canadensis	OR816	U.S.A.	Y	420	12/4/04	10/1/07	1030.7	2808
Ovis canadensis	OR817	U.S.A.	Y	420	12/4/04	5/27/07	903.7	2265
Ovis canadensis	WY806	U.S.A.	Y	420	3/19/04	6/1/07	1169.4	3263
Panthera leo	Diana	Kenya	Y	360	1/12/05	10/1/06	627.8	266
Panthera leo	Kiboche	Kenya	N	718	10/17/05	7/21/07	642	1465
Panthera leo	Romeo	Kenya	Y	420	4/27/02	9/13/02	138.2	396
Panthera onca	#223667461	Brazil	Y	21580	8/7/00	11/27/01	477	17
Panthera onca	#223670344	Brazil	Y	1440	7/16/02	8/4/02	19	18
Panthera onca	0-333005	Paraguay	N	480	6/15/10	3/8/11	265.4	288
Panthera onca	0-356798	Paraguay	Y	2235	7/21/10	12/9/11	505.5	150
Panthera onca	0-357643	Paraguay	Y	492	6/23/09	9/19/09	87.7	148
Panthera onca	103	Brazil	Y	660	3/22/05	4/10/06	383.8	797
Panthera onca	1111	Brazil	N	701	1/1/11	1/24/12	388	88
Panthera onca	11157	Brazil	Y	480	10/10/12	10/23/13	378.7	705
Panthera onca	11158	Brazil	Y	481	6/21/13	12/23/13	184.8	301
Panthera onca	11159	Brazil	Y	720	10/15/12	4/13/13	179.7	240
Panthera onca	11160	Brazil	N	481	6/10/13	10/13/13	125.2	167
Panthera onca	11161	Brazil	Y	480	6/18/13	8/28/13	70.8	109
Panthera onca	11162	Brazil	Y	960	10/22/13	1/29/14	99	133
Panthera onca	122154	Brazil	Y	360	1/17/15	7/26/15	190	42
Panthera onca	130780	Brazil	Y	360	10/21/13	11/8/14	383	295
Panthera onca	1494	Brazil	Y	120	11/21/12	1/23/14	427.9	3698

Continued on next page

Table S2.1 – continued from previous page

Species	Individual ID	Country	Consistent	Interval	Start Date	End Date	Duration	Locations
Panthera onca	152100	Brazil	Y	180	6/23/08	7/15/09	387.9	1645
Panthera onca	152150	Brazil	Y	180	7/5/08	10/22/09	474	2113
Panthera onca	152200	Brazil	N	180	7/17/08	4/26/11	1013.6	1758
Panthera onca	152201	Brazil	Y	180	6/20/08	3/10/09	262.9	951
Panthera onca	152250	Brazil	Y	180	7/25/08	11/6/08	104.4	481
Panthera onca	152300	Brazil	Y	180	7/30/08	12/8/08	131.5	287
Panthera onca	152350	Brazil	Y	360	2/2/10	5/13/10	99.8	166
Panthera onca	152351	Brazil	Y	180	8/11/08	7/15/09	338.1	709
Panthera onca	152600	Brazil	Y	180	9/24/09	10/29/11	765.6	227
Panthera onca	152601	Brazil	Y	180	7/16/08	10/14/08	89.7	202
Panthera onca	152650	Brazil	N	180	8/4/08	10/22/08	79.1	165
Panthera onca	2314	Brazil	Y	60	9/6/13	2/6/14	152.1	1633
Panthera onca	2315	Brazil	Y	210	4/24/15	10/20/15	179.1	799
Panthera onca	2323	Brazil	N	480	1/27/12	3/13/12	45.7	112
Panthera onca	2500	Argentina	Y	91	5/8/10	7/6/10	58.7	479
Panthera onca	27470	Argentina	Y	2700	2/19/13	6/27/13	128.3	53
Panthera onca	31935	Brazil	Y	59	11/1/11	12/23/11	52	1003
Panthera onca	31936	Brazil	Y	59	10/30/11	1/11/12	72.7	741
Panthera onca	32523	Brazil	Y	60	5/15/12	6/19/12	34.1	413
Panthera onca	325331	Paraguay	Y	240	7/4/11	12/1/11	149.3	500
Panthera onca	32781	Mexico	N	120	1/12/14	12/3/15	690.7	979
Panthera onca	32846	Brazil	Y	60	10/22/12	12/14/12	52.6	876
Panthera onca	32847	Brazil	Y	60	4/21/13	1/21/14	275	4860
Panthera onca	33299	Brazil	Y	60	10/19/13	1/3/14	76	1323
Panthera onca	33300	Brazil	Y	60	10/22/13	1/17/14	87.5	1391
Panthera onca	34691	Brazil	Y	62	10/9/13	4/20/14	193.3	1296
Panthera onca	35956	Brazil	Y	60	11/14/14	1/10/15	57.2	1361
Panthera onca	35957	Brazil	Y	60	10/12/15	2/15/16	126	2820
Panthera onca	35959	Brazil	Y	60	10/11/15	4/18/16	189.4	3339
Panthera onca	36112	Brazil	Y	60	4/26/15	8/30/15	126.7	2343
Panthera onca	36113	Brazil	Y	60	4/20/15	8/25/15	127.5	2300
Panthera onca	36313	Brazil	Y	60	12/5/14	8/17/15	255	4951
Panthera onca	36315	Brazil	Y	60	12/7/14	8/24/15	259.8	5038
Panthera onca	36316	Brazil	Y	60	11/28/14	12/25/14	27.3	615
Panthera onca	36317	Brazil	Y	60	11/29/14	4/13/15	134.9	2313
Panthera onca	36318	Brazil	Y	60	9/11/14	5/21/15	252	4708
Panthera onca	497364	Paraguay	N	240	8/9/05	8/19/06	374.6	1694
Panthera onca	497364C	Paraguay	Y	242	7/25/08	7/2/09	341.5	798
Panthera onca	5	Brazil	Y	360	2/17/13	7/19/13	151.2	491
Panthera onca	50886	Paraguay	Y	180	7/16/11	11/18/11	124.9	621
Panthera onca	535482	Paraguay	Y	140	6/26/04	9/16/04	81.8	620
Panthera onca	535483B	Paraguay	Y	240	6/29/06	5/21/07	326.2	1376
Panthera onca	62942700	Brazil	Y	60	9/16/15	6/2/16	260	5589
Panthera onca	655551A	Mexico	Y	580	1/17/12	9/14/13	605.8	104
Panthera onca	655553A	Mexico	Y	580	1/8/12	2/24/13	413.5	635
Panthera onca	655555A	Mexico	Y	581	8/25/12	11/10/13	442	443
Panthera onca	655556A	Guatemala	Y	291	8/14/12	12/24/12	131.9	72
Panthera onca	670290B	Paraguay	Y	121	6/29/14	12/31/14	184.6	1018
Panthera onca	670292B	Paraguay	Y	121	6/26/14	5/16/15	323.5	1667
Panthera onca	670294A	Paraguay	Y	240	7/12/14	7/9/15	361.3	913
Panthera onca	7272	Brazil	Y	240	9/21/10	11/6/10	46.2	133
Panthera onca	7273	Brazil	Y	241	7/12/10	8/26/10	45.2	129
Panthera onca	7274	Brazil	N	479	7/8/10	10/14/10	97.3	205
Panthera onca	8977	Brazil	N	1200	7/2/11	8/20/11	49.2	28
Panthera onca	8982	Brazil	Y	960	6/26/11	10/29/11	124.7	141
Panthera onca	8983	Brazil	N	719	7/8/11	10/4/11	88.5	109
Panthera onca	8984	Brazil	Y	1080	7/8/11	9/30/11	83.7	67
Panthera onca	8988	Brazil	Y	600	10/14/11	11/19/12	402.2	581
Panthera onca	8991	Brazil	Y	1680	10/15/11	5/13/12	211.6	133
Panthera onca	8993	Brazil	Y	961	10/22/13	2/1/14	102.2	103
Panthera onca	919458	Brazil	Y	420	8/27/15	3/29/16	215.8	404
Panthera onca	921766	Brazil	Y	241	3/29/16	5/23/16	55.4	151
Panthera onca	921790	Brazil	N	360	6/13/14	6/19/14	6.3	20
Panthera onca	921941	Brazil	Y	300	8/22/15	6/24/16	306.6	134
Panthera onca	Buck	Paraguay	Y	180	4/21/11	8/12/12	479.3	3461
Panthera onca	D563517	Paraguay	Y	718	8/3/11	6/3/12	304.7	327
Panthera onca	F1	Brazil	N	1441	2/11/03	7/3/03	142	92
Panthera onca	H 51	Costa Rica	Y	120	11/20/14	6/18/16	576.5	5922
Panthera onca	MIREYLLE	Brazil	Y	292	3/1/14	10/31/14	244.3	1010
Panthera onca	NATALIA	Brazil	Y	484	3/7/14	6/1/14	85.3	131
Panthera onca	No1	Paraguay	Y	280	6/21/02	6/28/03	372.2	1094
Panthera onca	No2	Paraguay	Y	280	7/16/03	7/29/04	379	921
Panthera onca	No3	Paraguay	N	281	7/18/03	7/15/04	362.8	722
Panthera onca	No5	Paraguay	Y	240	8/9/05	5/6/06	269.9	1300
Panthera onca	TIAGO	Brazil	Y	314	3/12/14	2/13/15	337.1	780
Panthera pardus pardus	Pp F CH	Kenya	Y	15	1/26/14	5/25/14	118.5	6872
Panthera pardus pardus	Pp F HA	Kenya	Y	15	1/24/14	1/19/15	360	25290
Panthera pardus pardus	Pp F KO	Kenya	Y	15	1/20/14	1/27/15	371.7	21166
Panthera pardus saxicolor	F5_Iran	Iran	Y	60	12/7/15	2/6/16	61.5	884
Panthera pardus saxicolor	M1_Borzou	Iran	N	60	2/9/15	2/6/16	361.6	5152
Panthera pardus saxicolor	M2_Bardia	Iran	Y	180	10/3/14	9/30/15	362.6	3632
Panthera pardus saxicolor	M3_Borna	Iran	Y	61	9/28/14	9/28/15	364.2	4153
Panthera pardus saxicolor	M4_Tandoureh	Iran	Y	60	8/16/16	4/2/17	228.8	2608
Papio anubis	Pa AI WG	Kenya	N	15	1/19/14	10/10/14	263.8	22961
Papio anubis	Pa AI YK	Kenya	Y	15	1/19/14	1/15/15	360.9	33114
Papio anubis	Pa LI LU	Kenya	Y	15	1/15/14	6/29/14	165.3	15631
Papio anubis	Pa LI TH	Kenya	Y	15	1/15/14	6/8/14	144.4	13622
Papio anubis	Pa MG SH	Kenya	Y	15	1/23/14	1/8/15	349.8	30234

Continued on next page

Table S2.1 – continued from previous page

Species	Individual ID	Country	Consistent	Interval	Start Date	End Date	Duration	Locations
Papio anubis	Pa ST MS	Kenya	Y	15	1/16/14	1/27/15	376.3	35542
Papio cynocephalus	1	Kenya	N	60	3/20/08	1/11/09	297.5	4127
Papio cynocephalus	2	Kenya	Y	60	11/8/09	9/3/10	299.5	4140
Papio cynocephalus	3	Kenya	Y	60	11/15/09	9/8/10	297.5	4099
Papio cynocephalus	4	Kenya	N	60	3/12/06	6/7/06	87.5	1222
Papio cynocephalus	5	Kenya	Y	60	3/16/08	1/8/09	298.5	4113
Papio cynocephalus	6	Kenya	Y	60	11/6/09	8/28/10	295.5	4107
Papio cynocephalus	7	Kenya	N	60	11/13/09	9/7/10	298.5	4102
Papio cynocephalus	8	Kenya	Y	60	1/8/09	10/29/09	294.5	4098
Papio cynocephalus	9	Kenya	Y	60	1/9/09	10/31/09	295.5	4098
Papio cynocephalus	10	Kenya	Y	60	11/4/09	8/24/10	293.5	4059
Papio cynocephalus	11	Kenya	Y	60	3/21/10	9/18/10	181.5	2498
Papio cynocephalus	12	Kenya	Y	60	12/3/08	9/26/09	297.5	3788
Papio cynocephalus	13	Kenya	Y	60	3/14/08	1/5/09	297.5	4113
Papio cynocephalus	14	Kenya	Y	60	1/9/09	10/30/09	294.5	4072
Papio cynocephalus	15	Kenya	N	60	11/11/09	9/1/10	294.5	4072
Papio cynocephalus	16	Kenya	Y	60	3/13/09	11/3/09	235.5	3286
Papio cynocephalus	17	Kenya	N	60	3/22/08	1/13/09	297.5	4091
Papio cynocephalus	18	Kenya	N	60	11/13/09	9/6/10	297.5	4095
Papio cynocephalus	19	Kenya	N	60	11/10/09	9/3/10	297.5	4113
Papio cynocephalus	21	Kenya	Y	60	1/11/09	11/4/09	297.5	4078
Papio cynocephalus	22	Kenya	Y	60	12/5/08	9/28/09	297.5	4104
Pecari tajacu	Merk 4665	Panama	Y	0.02	3/2/16	3/31/16	28.9	25640
Pecari tajacu	Grupo	Argentina	N	83	5/8/15	8/18/16	468	381
Pecari tajacu	Sayky	Argentina	Y	124	6/18/15	8/16/15	59.1	65
Potos flavus	Abby 4652	Panama	Y	0	12/15/15	4/1/16	107.2	94306
Potos flavus	Ben Bob 4653	Panama	N	0	12/15/15	3/4/16	79.5	49860
Potos flavus	Bonnie 4658	Panama	Y	0	12/15/15	4/18/16	124.4	92154
Potos flavus	Chloe 4052	Panama	Y	0	12/15/15	1/3/16	18.2	14368
Potos flavus	Eli 5765	Panama	Y	0	12/15/17	3/4/18	79.6	76340
Potos flavus	Gamer 5772	Panama	Y	0	12/15/17	2/15/18	62	59591
Potos flavus	Jeff 5769	Panama	Y	0	12/15/17	2/22/18	68.1	61443
Potos flavus	Judy 4656	Panama	Y	0	1/28/16	3/12/16	44.5	40335
Potos flavus	Mario 5768	Panama	Y	0	12/15/17	2/25/18	72	65860
Potos flavus	Molly 5770	Panama	N	0	12/15/17	2/14/18	60.7	56482
Potos flavus	Ripley 4650	Panama	Y	0	12/15/15	4/7/16	113	71340
Potos flavus	Ripley 5771	Panama	N	0	12/15/17	2/22/18	68.1	66983
Potos flavus	Tony Stark 4659	Panama	Y	0	12/15/17	2/19/18	65.4	63592
Procyon lotor	B1	Japan	Y	60	12/1/10	1/27/11	56.9	691
Procyon lotor	B2	Japan	Y	60	2/9/11	3/7/11	26.2	317
Procyon lotor	F1	Japan	Y	31	7/9/12	7/31/12	22.6	254
Procyon lotor	M1_Winter	Japan	Y	31	2/29/12	3/15/12	14.6	338
Procyon lotor	M2	Japan	N	30	11/9/12	11/29/12	19.8	268
Procyon lotor	M3	Japan	Y	30	11/21/12	12/1/12	9.7	157
Procyon lotor	Hector	U.S.A.	Y	15	9/4/13	10/7/13	32.6	826
Procyon lotor	Heidi	U.S.A.	Y	15	9/4/13	10/4/13	30.2	1844
Propithecus verreauxi	128ee	Madagascar	Y	15	9/4/12	10/26/12	51.7	4755
Propithecus verreauxi	129c	Madagascar	Y	15	9/4/12	12/9/12	95.7	8790
Propithecus verreauxi	2075j	Madagascar	Y	15	3/23/12	7/18/12	116.7	11287
Propithecus verreauxi	2076f	Madagascar	Y	15	3/22/12	7/11/12	110.4	5898
Propithecus verreauxi	2077c	Madagascar	Y	15	3/22/12	8/7/12	137.5	13352
Propithecus verreauxi	2078g	Madagascar	Y	15	3/22/12	8/7/12	137.7	13326
Propithecus verreauxi	2079ee	Madagascar	Y	15	3/22/12	8/19/12	149.5	6039
Propithecus verreauxi	2519ee	Madagascar	Y	15	12/11/12	1/12/13	31.8	3062
Propithecus verreauxi	2523j	Madagascar	Y	15	8/21/13	11/28/13	98.8	9473
Propithecus verreauxi	2525f1	Madagascar	Y	15	1/29/13	5/2/13	92.7	8914
Propithecus verreauxi	2527g	Madagascar	Y	15	8/21/13	12/4/13	104.7	10118
Propithecus verreauxi	2528j	Madagascar	Y	15	3/13/13	7/31/13	139.7	13535
Propithecus verreauxi	2531ee	Madagascar	Y	15	3/15/13	5/8/13	53.8	4926
Propithecus verreauxi	2532c	Madagascar	Y	15	1/15/13	5/1/13	105.8	10269
Propithecus verreauxi	2533f	Madagascar	Y	15	10/25/12	1/13/13	79.7	7518
Propithecus verreauxi	2534j	Madagascar	Y	15	10/25/12	1/9/13	75.8	7233
Propithecus verreauxi	2535f	Madagascar	Y	15	3/5/13	5/6/13	61.7	5966
Propithecus verreauxi	2536h	Madagascar	Y	15	12/12/12	4/11/13	119.8	11549
Propithecus verreauxi	2723g	Madagascar	Y	15	4/16/13	8/22/13	127.8	12213
Propithecus verreauxi	2808ee	Madagascar	Y	15	8/20/13	8/28/13	7.7	701
Propithecus verreauxi	2809h	Madagascar	Y	15	4/16/13	8/23/13	128.7	12472
Propithecus verreauxi	2810f	Madagascar	Y	15	8/21/13	12/2/13	102.7	9867
Propithecus verreauxi	2811c	Madagascar	Y	15	8/20/13	10/25/13	65.8	6345
Propithecus verreauxi	2812h	Madagascar	Y	15	8/22/13	12/6/13	105.8	10140
Propithecus verreauxi	2814ee	Madagascar	Y	15	9/10/13	12/6/13	86.8	8336
Propithecus verreauxi	2817l	Madagascar	Y	15	8/22/13	12/6/13	105.7	10155
Propithecus verreauxi	2818f1	Madagascar	Y	15	8/20/13	10/25/13	65.7	6347
Propithecus verreauxi	562f1	Madagascar	Y	15	8/14/12	11/13/12	90.5	8306
Sus scrofa	150011	Brazil	Y	4	4/19/12	6/15/12	56.7	11120
Sus scrofa	150041	Brazil	Y	5	4/18/12	6/15/12	58	7506
Sus scrofa	150102	Brazil	Y	5	4/17/12	6/14/12	57.4	4650
Sus scrofa	150192	Brazil	Y	4	4/18/12	5/13/12	25.4	6925
Sus scrofa	150281	Brazil	Y	5	4/18/12	6/14/12	56.8	5985
Sus scrofa	150312	Brazil	Y	5	4/18/12	6/12/12	55.2	5366
Sus scrofa	150462	Brazil	N	11	4/18/12	6/14/12	56.6	4615
Sus scrofa	150492	Brazil	Y	4	4/17/12	6/14/12	57.7	12064
Sus scrofa	150522	Brazil	Y	5	4/19/12	6/16/12	57.6	6796
Sus scrofa	150552	Brazil	Y	5	4/19/12	6/15/12	56.7	11009
Sus scrofa	150681	Brazil	Y	10	4/20/12	6/17/12	58.1	4961
Sus scrofa	150711	Brazil	N	4	4/19/12	6/18/12	59.6	13303
Sus scrofa	164466	Brazil	Y	4	4/20/12	6/16/12	57	12521
Sus scrofa	164509	Brazil	N	4	4/20/12	6/16/12	57	12310

Continued on next page

Table S2.1 – continued from previous page

Species	Individual ID	Country	Consistent	Interval	Start Date	End Date	Duration	Locations
Sus scrofa	164541	Brazil	Y	4	4/21/12	6/16/12	55.9	12207
Sus scrofa	164590	Brazil	N	4	4/19/12	5/22/12	33.1	4437
Sus scrofa	164820	Brazil	Y	15	3/9/12	4/13/12	35.2	1812
Sus scrofa	164860	Brazil	Y	4	4/20/12	6/16/12	56.7	6079
Sus scrofa	164900	Brazil	N	5	4/21/12	6/17/12	57.1	11821
Sus scrofa	Briga	Brazil	N	60	9/28/11	12/13/11	76.1	1210
Sus scrofa	Mae	Brazil	N	60	7/26/11	9/3/11	39.2	465
Sus scrofa	Moda_queixada	Brazil	N	60	5/28/11	11/22/11	178.1	2566
Sus scrofa	Mordida	Brazil	Y	60	4/17/11	6/13/11	57.4	442
Sus scrofa	Negao	Brazil	Y	60	4/16/11	6/1/11	45.9	688
Sus scrofa	Russao	Brazil	Y	60	4/18/11	5/18/11	30.2	516
Sus scrofa	Velho	Brazil	N	60	4/15/11	11/14/11	213.4	2612
Syncerus caffer	Cilla	South Africa	Y	60	7/14/05	12/7/05	146.7	3527
Syncerus caffer	Gabs	South Africa	Y	60	4/5/05	6/27/05	82.9	1996
Syncerus caffer	Mvubu	South Africa	Y	60	7/15/05	10/29/05	106.6	2572
Syncerus caffer	Pepper	South Africa	Y	120	4/25/06	12/31/06	250.4	1725
Syncerus caffer	Queen	South Africa	Y	60	2/17/05	6/2/05	104.9	1756
Syncerus caffer	Toni	South Africa	Y	60	8/23/05	4/22/06	242.7	5766
Tolypeutes matacus	tm14	Brazil	Y	5	1/6/07	1/6/18	4019	5396
Tolypeutes matacus	TM15	Brazil	Y	5	1/6/07	1/6/18	4019	8248
Tolypeutes matacus	TM16	Brazil	Y	5	1/5/12	1/4/17	1826.2	1197
Tolypeutes matacus	TM17	Brazil	Y	5	1/6/07	1/6/18	4018.9	4420
Tolypeutes matacus	TM21	Brazil	Y	5	2/6/07	2/6/18	4019	4664
Tolypeutes matacus	Tm24	Brazil	Y	5	1/6/07	1/6/18	4019	3705
Tolypeutes matacus	Tm30	Brazil	Y	5	1/7/07	2/5/18	4048	5056
Tolypeutes matacus	Tm33	Brazil	Y	5	2/7/07	2/6/18	4018	2632
Tolypeutes matacus	tm35	Brazil	Y	5	2/6/07	2/6/18	4019	4841
Tolypeutes matacus	tm37	Brazil	Y	5	2/6/07	2/6/18	4019	4408
Ursus americanus	BB03	U.S.A.	Y	15	6/21/09	9/22/09	92.1	8608
Ursus americanus	BB05	U.S.A.	Y	15	6/29/09	9/20/09	82.8	7805
Ursus americanus	BB06	U.S.A.	Y	15	6/30/09	9/18/09	79.9	7409
Ursus americanus	BB07	U.S.A.	Y	15	7/1/09	9/6/09	66.5	6078
Ursus americanus	BB08	U.S.A.	N	15	7/11/09	6/30/11	718.6	15594
Ursus americanus	BB09	U.S.A.	N	15	7/12/09	12/3/09	143.9	13257
Ursus americanus	BB10	U.S.A.	Y	15	7/13/09	8/11/09	29	2735
Ursus americanus	BB11	U.S.A.	Y	15	7/19/09	9/27/09	69.9	6485
Ursus americanus	BB12	U.S.A.	N	15	7/21/09	12/3/09	135	11179
Ursus americanus	BB14	U.S.A.	N	15	8/4/09	12/3/09	121	9598
Ursus americanus	BB16	U.S.A.	Y	15	2/10/11	5/23/11	101.2	1326
Ursus americanus	BB28	U.S.A.	Y	15	1/15/11	9/10/11	237.5	12323
Ursus americanus	BB39	U.S.A.	Y	15	1/1/11	5/20/11	139.1	2025
Ursus americanus	BB44	U.S.A.	Y	15	12/17/10	8/18/11	243.5	10252
Ursus americanus	BB53	U.S.A.	Y	15	5/25/11	9/9/11	106.6	7650
Ursus americanus	BB54	U.S.A.	Y	14	5/29/11	9/11/11	104.3	10031
Ursus americanus	BB58	U.S.A.	Y	15	6/7/11	12/7/11	182.8	13433
Ursus arctos	Aga	Poland	Y	30	10/28/14	10/9/15	345.6	13423
Ursus arctos	Babros	Poland	Y	241	4/25/07	6/8/07	44.2	182
Ursus arctos	Cyrhla	Poland	N	30	5/20/14	5/29/15	373.6	266
Ursus arctos	Djuro	Ukraine	Y	30	11/9/14	12/23/14	44.8	2112
Ursus arctos	Emil	Poland	Y	30	3/25/15	10/8/15	196.9	4269
Ursus arctos	Eryk	Slovakia	Y	122	5/18/14	5/8/15	354.7	2016
Ursus arctos	Filip	Slovakia	Y	120	4/11/14	1/12/15	275.6	824
Ursus arctos	Nestor	Poland	Y	478	4/20/08	5/17/08	26.8	62
Ursus arctos	Nuria	Poland	Y	30	3/20/15	9/26/15	189.7	5725
Ursus arctos	Roma	Poland	Y	30	10/10/15	10/19/15	8.4	403
Ursus arctos	Roztoka	Slovakia	Y	241	10/20/06	11/4/07	379.8	825
Ursus arctos	Valer	Slovakia	Y	61	4/21/13	12/29/13	251.8	2164
Vulpes bengalensis	Fox 03	India	N	61	12/5/15	2/11/16	68.3	850
Vulpes bengalensis	Fox 05	India	Y	61	9/8/15	10/2/15	24	418
Vulpes bengalensis	Fox 08	India	N	179	10/17/16	7/10/17	265.5	2617
Vulpes bengalensis	Fox 10	India	Y	180	11/13/16	6/25/17	223.9	1272
Vulpes bengalensis	Fox 12	India	Y	179	1/6/17	6/6/17	150.4	1586
Vulpes bengalensis	Fox 13	India	Y	179	1/8/17	7/25/17	197.6	2039
