# Supplementary material for: Effects of body size on estimation of mammalian area requirements
Source: Conserv Biol. 2020 Jun 18;34(4):1017–28. doi: 10.1111/cobi.13495 (PMC7496598; doi:10.1111/cobi.13495)
Supplement: Supplementary file 3 — Supplementary Material [file COBI-34-1017-s003.pdf]

Supporting Information S3 — Mammalian phylogenetic relationships

Details on the phylogenetic relationships between species analyzed in the main text.

Order Carnivora

Phylogenetic relationships for the Carnivora were taken from a consensus phylogeny (Agnarsson et al., 2010). Agnarsson et al. (2010) did not include the Bengal fox (*Vulpes bengalensis*), nor the crab-eating fox (*Cerdocyon thous*) in their comparison. Consequently, for the Canidae, we took relationships from Lindblad-Toh et al. (2005), due to its inclusion of all of the canid species for which we had tracking data. The resulting phylogeny is depicted in figure S3.1.

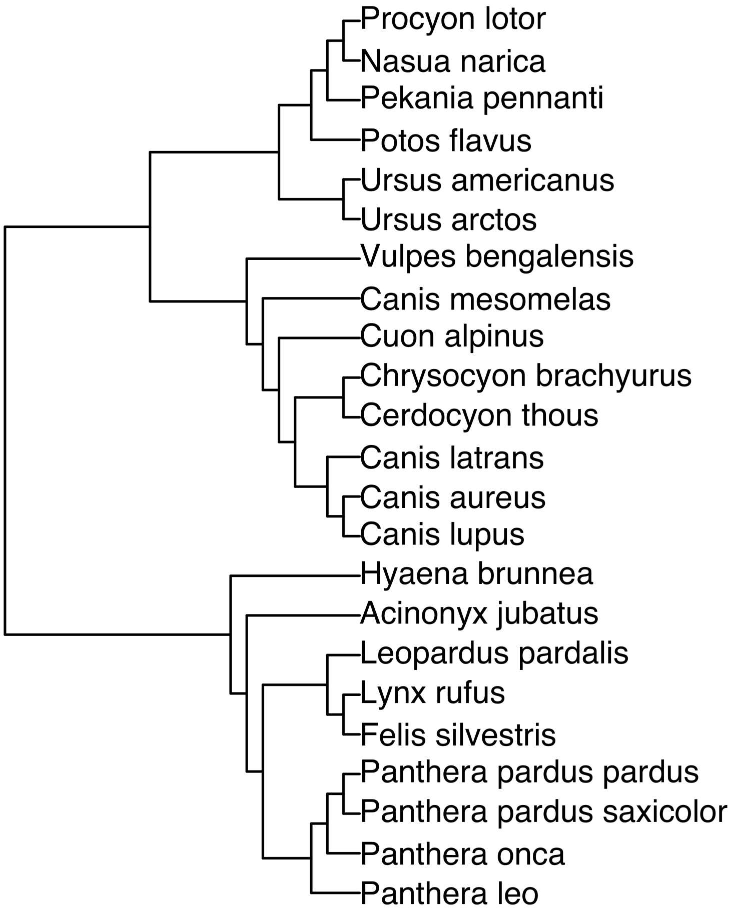

Figure S3.1: Phylogenetic relationships among the Carnivora species analyzed in the main text.

Order Perissodactyla

Phylogenetic relationships for the Perissodactyla were taken from Steiner & Ryder (2011). The relationships between the three species for which we had tracking data are depicted in figure S3.2.

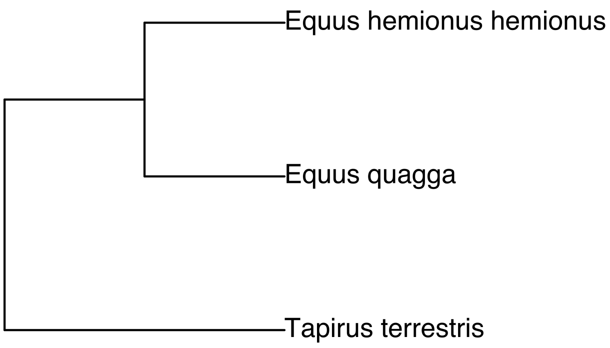

Figure S3.2: Phylogenetic relationships among the Perissodactyla species analyzed in the main text.

Order Primates

Phylogenetic relationships between the Primates were taken from (Perelman et al., 2011). The relationships between those species for which we had tracking data are depicted in figure S3.3.

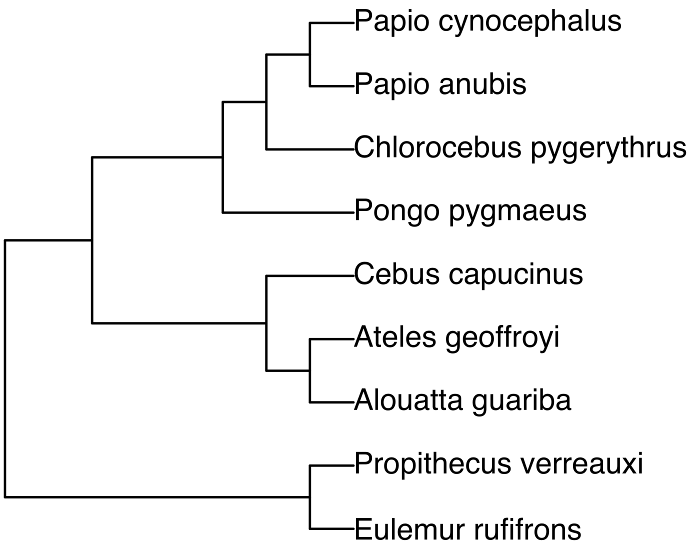

Figure S3.3: Phylogenetic relationships among the Primates species analyzed in the main text.

Order Artiodactyla

Phylogenetic relationships for the Artiodactyla were taken from Price et al. (2005). The relationships between those species for which we had tracking data are depicted in figure S3.4.

Order Lagomorpha

Phylogenetic relationships for the Lagomorpha were taken from Matthee et al. (2004). The relationships between the three species for which we had tracking data are depicted in figure S3.5.

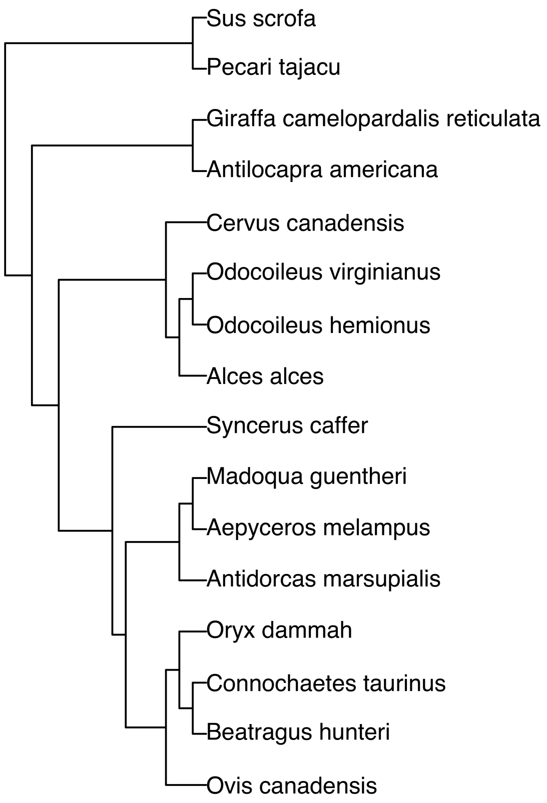

Figure S3.4: Phylogenetic relationships among the Artiodactyla species analyzed in the main text.

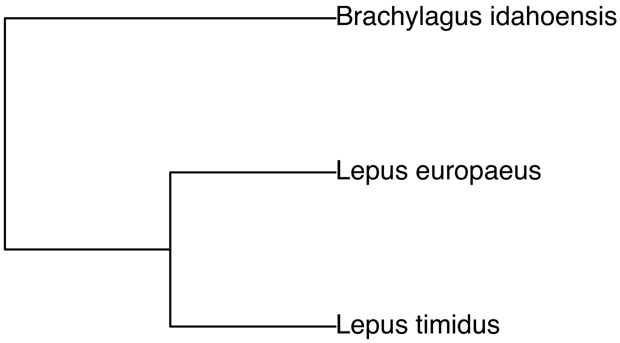

Figure S3.5: Phylogenetic relationships among the Lagomorpha species analyzed in the main text.

Order Proboscidea

Phylogenetic relationships for the Proboscidea were taken from Barriel et al. (1999). The relationships between the three species for which we had tracking data are depicted in figure S3.6.

Order Eulipotyphla

We only had tracking data from one species from the order Eulipotyphla, the European hedgehog (*Erinaceus europaeus*). Consequently, no intra Order relationships were necessary.

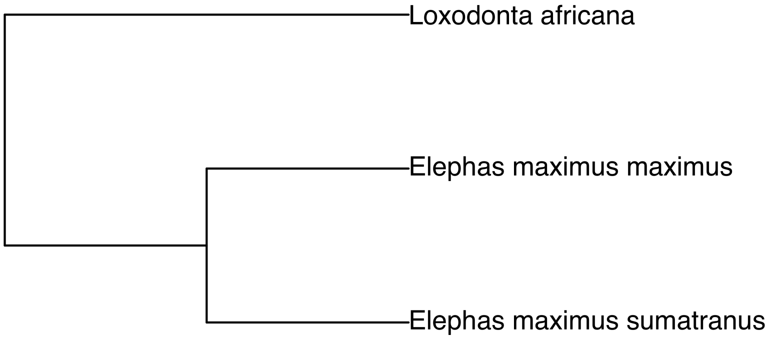

Figure S3.6: Phylogenetic relationships among the Proboscidea species analyzed in the main text.

Order Cingulata

We only had tracking data from two species from the order Cingulata, the six-banded armadillo (*Euphractus sexcinctus*) and the Southern three-banded armadillo (*Tolypeutes matacus*). As such, no intra Order relationships were necessary here.

Order Rodentia

We only had tracking data from one species from the order Rodentia, the Central American agouti (*Dasyprocta punctata*). Consequently, no intra Order relationships were necessary.

Order Marsupialia

We only had tracking data from one species from the order Marsupialia, the Virginia opossum (*Didelphis virginiana*), and so no intra Order relationships were necessary.

Class Mammalia

After generating the order specific trees, we used the phylogenetic relationships between eutherian mammalian orders from Liu et al. (2001) to assemble the complete phylogenetic tree. The complete phylogenetic tree for the 61 Mammalian species for which we had tracking data is depicted in figure S3.7.

References

Agnarsson, I., M. Kuntner and L. J. May-Collado, 2010. Dogs, cats, and kin: a molecular species-level phylogeny of Carnivora. Molecular Phylogenetics and Evolution **54**(3):726–745.

Barriel, V., E. Thuet and P. Tassy, 1999. Molecular phylogeny of Elephantidae. Extreme divergence of the extant forest African elephant. Comptes Rendus de l'Académie des Sciences-Series III-Sciences de la Vie **322**(6):447–454.

Lindblad-Toh, K., C. M. Wade, T. S. Mikkelsen, E. K. Karlsson, D. B. Jaffe, M. Kamal, M. Clamp, J. L. Chang, E. J. Kulbokas, M. C. Zody et al., 2005. Genome sequence, comparative analysis and haplotype structure of the domestic dog. Nature **438**(7069):803–819.

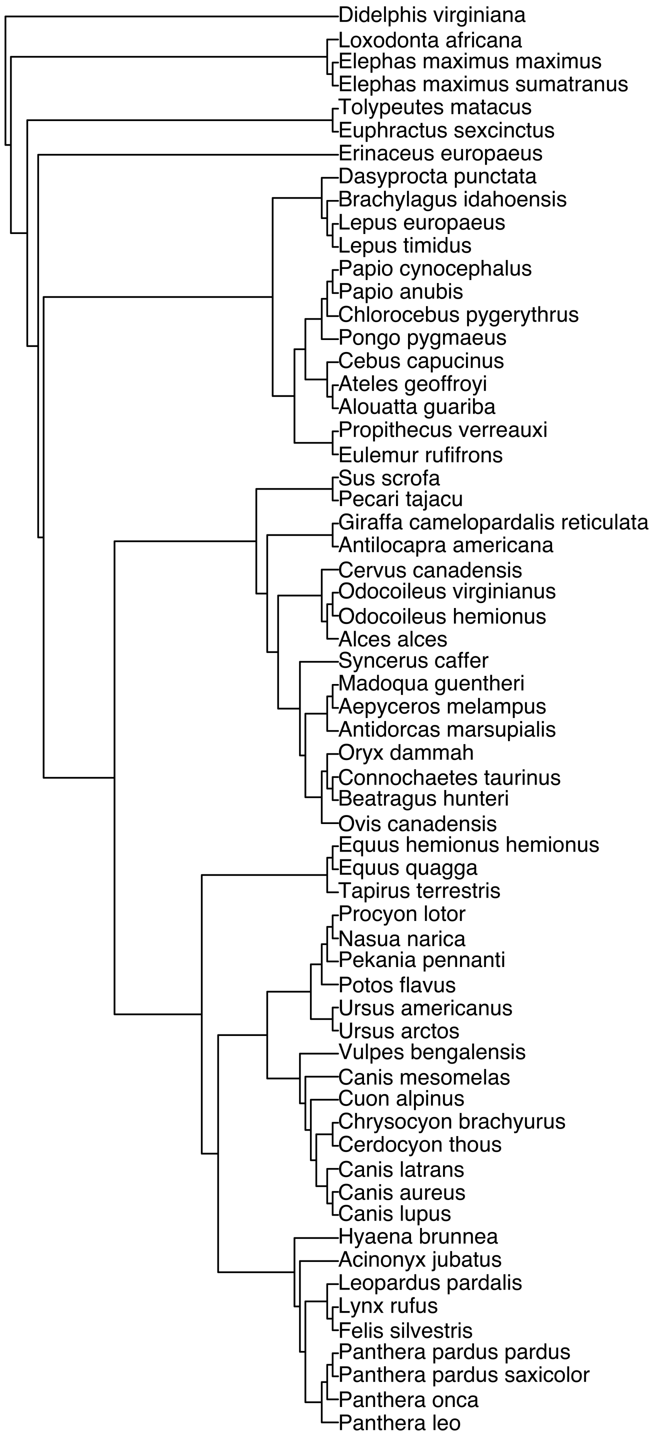

Figure S3.7: Phylogenetic relationships among the Mammalian species analyzed in the main text.

Liu, F.-G. R., M. M. Miyamoto, N. P. Freire, P. Q. Ong, M. R. Tennant, T. S. Young and K. F. Gugel, 2001. Molecular and morphological supertrees for eutherian (placental) mammals. Science **291**(5509):1786–1789.

Matthee, C. A., B. J. van Vuuren, D. Bell and T. J. Robinson, 2004. A molecular supermatrix of

the rabbits and hares (Leporidae) allows for the identification of five intercontinental exchanges during the Miocene. Systematic Biology **53**(3):433–447.

Perelman, P., W. E. Johnson, C. Roos, H. N. Seuánez, J. E. Horvath, M. A. M. Moreira, B. Kessing, J. Pontius, M. Roelke, Y. Rumpler et al., 2011. A molecular phylogeny of living primates. PLOS Genetics **7**(3):e1001342.

Price, S. A., O. R. P. Bininda-Emonds and J. L. Gittleman, 2005. A complete phylogeny of the whales, dolphins and even-toed hoofed mammals (Cetartiodactyla). Biological Reviews of the Cambridge Philosophical Society **80**(3):445–473.

Steiner, C. C. and O. A. Ryder, 2011. Molecular phylogeny and evolution of the Perissodactyla. Zoological Journal of the Linnean Society **163**(4):1289–1303.
